# Superlattice Engineering on 2D Bi_2_Te_3_‐Sb_2_Te_3_ Chalcogenides

**DOI:** 10.1002/advs.202503492

**Published:** 2025-04-07

**Authors:** Han Wang, Songqing Zhang, Huijia Luo, Wenwu Pan, Zekai Zhang, Junliang Liu, Yongling Ren, Cailei Yuan, Wen Lei

**Affiliations:** ^1^ Department of Electrical Electronic and Computer Engineering The University of Western Australia Crawley WA 6009 Australia; ^2^ School of Integrated Circuits Jiangnan University Wuxi 214122 China; ^3^ Jiangxi Key Laboratory of Nanomaterials and Sensors School of Physics Communication and Electronics Jiangxi Normal University 99 Ziyang Avenue Nanchang 330022 China

**Keywords:** 2D chalcogenide nanostructures, chemical vapor deposition, controllable growth, density functional theory, lateral and vertical superlattices

## Abstract

As a focal point in materials science, 2D superlattices, comprising periodically arranged nanostructures, have emerged as promising platforms for engineering, optoelectronic, and quantum phenomena. In this work, an innovative approach is studied to fabricate multi‐layered 2D Bi_2_Te_3_‐Sb_2_Te_3_ chalcogenide superlattices including wrapped, lateral and vertical ones. Using a novel precursor switching method, wrapped 2D van der Waals superlattices are synthesized via chemical vapor deposition. Thermal annealing and focused ion beam techniques are employed to achieve both lateral and vertical superlattices with controlled dimensions and sharp interfaces. Comprehensive structural and electronic characterization revealed the high crystalline quality and electronic properties of these superlattices. A comprehensive growth model is also developed to elucidate the growth mechanism and optimize the growth parameters. This research demonstrates a feasible approach to fabricate wrapped, lateral, and vertical superlattices, laying the foundation for further advanced study of their physical properties and potential device applications.

## Graphical abstract

This study presents an innovative approach to fabricating 2D Bi_2_Te_3_‐Sb_2_Te_3_ chalcogenide superlattices in wrapped, lateral, and vertical configurations. Utilizing a novel precursor switching method, chemical vapor deposition, and post‐growth techniques, the research achieves controlled superlattices with sharp interfaces. Comprehensive characterization and growth modeling provide insights into the formation of mechanisms and properties, opening avenues for advanced studies and potential applications.

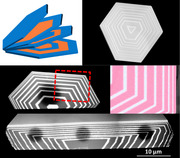

## 1 Introduction

Superlattices, as artificially engineered nanostructures, offer an attractive solution for tailoring the electronic, optical, and structural properties of materials.^[^ ^1^ ^]^ These periodic low‐dimensional structures consist of alternating layers of different materials (typically semiconductors or metals) arranged in a predictable arrangement.^[^ ^2^ ^]^ The ability to accurately control the chemical composition, sequence, and structural parameters of layers allows researchers to design the electronic band structures, carrier dynamics, and quantum confinement effects, leading to remarkable advancements in materials science and device engineering.^[^ ^3^ ^]^ In recent years, the achievement of superlattices at the atomic scale has provided new opportunities to explore novel physical phenomena and develop advanced applications in the fields of infrared photodetection, quantum computing, and energy harvesting.^[^ ^4^ ^]^ In comparison to traditional superlattices, superlattices based on 2D materials exhibit extra fascinating physical properties such as layer‐dependent band alignments,^[^ ^5^ ^]^ tunable moiré potentials,^[^ ^6^ ^]^ as well as intriguing interlayer interactions, such as charge transfer and excitonic effects^[^ ^7^ ^]^ due to the weak Van der Waals (vdW) force between 2D layers. This offers new opportunities for fabricating high‐performance electronic devices with tailored functionalities.^[^ ^8^ ^]^

As a typical class of vdW layered materials, narrow bandgap Sb_2_Te_3_, Bi_2_Te_3_, and Bi_2_Se_3_ chalcogenide materials have attracted significant attention due to their favorable physical properties. They are 3D topological insulators that present very high electron mobility and thus can lead to infrared photodetectors with high performance such as high quantum efficiency, fast response, etc.^[^ ^9^ ^]^ However, most of the photodetectors fabricated with these 2D layered materials are photoconductor devices based on individual single 2D materials, which, however, present a larger dark current and noise, and thus a lower detectivity.^[^ ^10^ ^]^ To achieve better device performance such as lower dark current and noise, and higher detectivity, it is essential to have heterostructures/superlattices with proper band structure design and doping like the state‐of‐the‐art photodiode detector.^[^ ^11^ ^]^ In addition, heterostructures/superlattices based on these chalcogenides will provide a platform for exploring intriguing physical properties and studying their applications in various electronic devices except photodetectors. However, it presents numerous challenges to have controlled growth of high‐quality superlattices based on these 2D chalcogenide materials, including lateral and vertical dimensions of the constituting layers, size and composition uniformity, and others. For example, as the number of layers and structural complexity increases, it becomes increasingly challenging to maintain high uniformity in terms of elemental composition and size. This challenge is exemplified by the fact that most vdW superlattices reported were fabricated via laborious micromechanical exfoliation and manual restacking procedures.^[^ ^12^ ^]^ These exfoliation and restacking processes suffer the issues of contamination, scalability, and reproducibility,^[^ ^13^ ^]^ making them suitable for ultimate industry manufacturing. Recently, some progress has been achieved in the synthesis of vdW heterostructures and superlattices^[^ ^14^ ^]^ through methods such as solution‐based synthesis^[^ ^15^ ^]^ and chemical vapor deposition (CVD).^[^ ^16^ ^]^ However, the synthesis processes reported are limited to simple two‐layer heterostructure and/or core‐shell multi‐layer structures and lack control over the heterostructure types (lateral or vertical ones) and dimensions (layer thickness and lateral size). For ultimate industry applications, it is essential to fabricate 2D vdW heterostructures and superlattices with precise control over heterostructure types and dimensions, which will benefit the design and fabrication of functional electronic devices.

**Figure 1:**
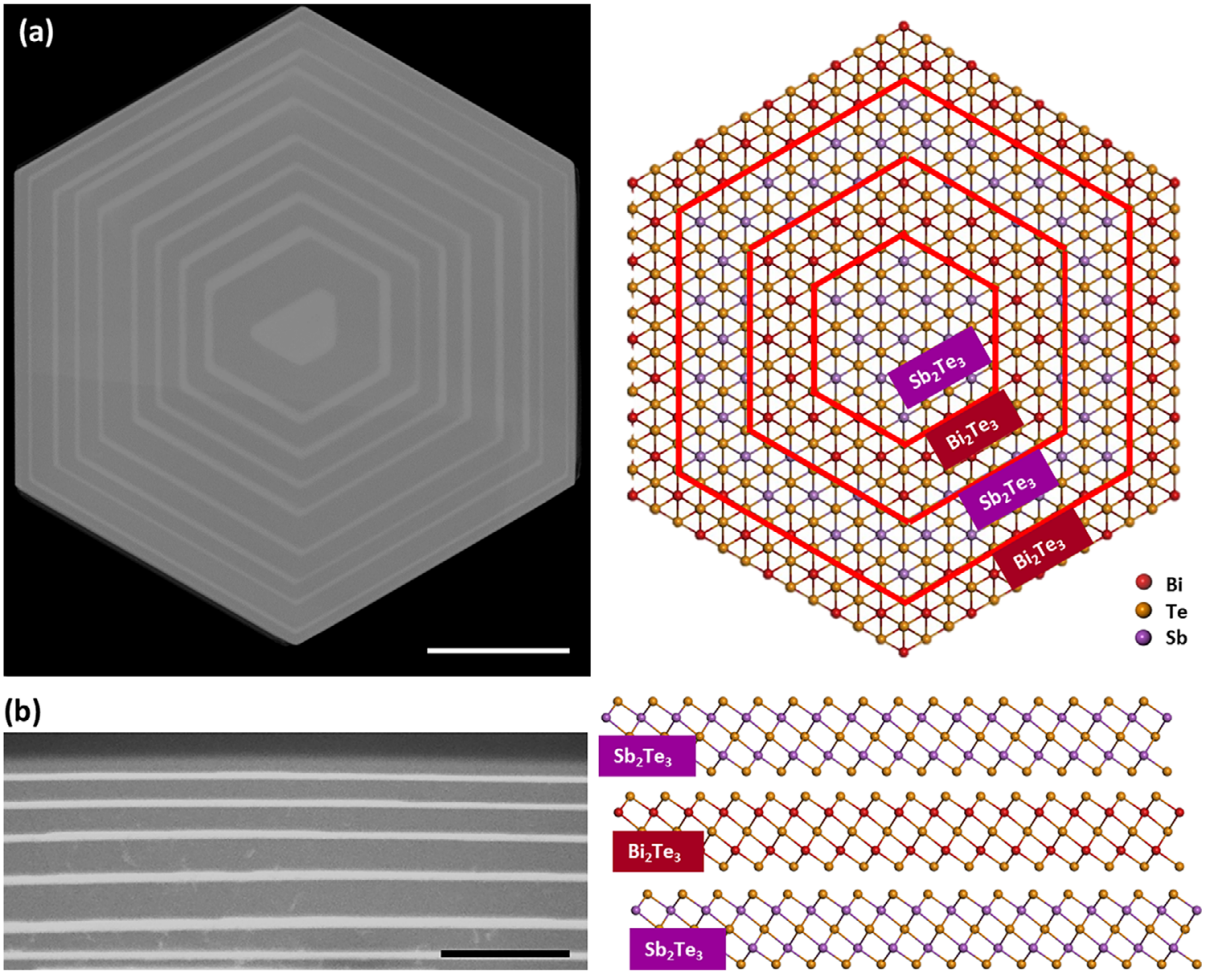
Lattice structures of 2D vdW superlattices in this work. a) Top‐view SEM image of a representative Bi_2_Te_3_‐Sb_2_Te_3_ lateral superlattices with 18 layers and their theoretical schematic lattice structures. The scale bar is 10 µm. b) Side‐view SEM image of a representative Bi_2_Te_3_‐Sb_2_Te_3_ vertical superlattices with 13 layers and their theoretical schematic lattice structures. The scale bar is 5 µm. Note that the dark field region is Sb_2_Te_3,_ while the bright field region is Bi_2_Te_3_.

Herein, this work presents an innovative study on the superlattice engineering of 2D Bi_2_Te_3_‐Sb_2_Te_3_ chalcogenides. Density functional theory (DFT) calculations were first undertaken on the interface energies between Bi_2_Te_3_ and Sb_2_Te_3_ binary alloys to understand and predict the formation and stability of Bi_2_Te_3_‐Sb_2_Te_3_ vdW superlattices. Wrapped 2D vdW structures integrated both lateral and vertical superlattices simultaneously, were achieved by implementing CVD growth with alternating precursor materials and optimizing growth conditions. These CVD‐grown superlattices demonstrated high material quality in terms of both structural and electronic quality. A growth model was also introduced to understand the growth mechanism and fabrication process which allows a better control over the 2D vdW superlattice growth and fabrication. Subsequent thermal decomposition and/or focused ion beam (FIB) milling/cutting were used to engineer the wrapped superlattices to achieve both lateral and vertical Bi_2_Te_3_‐Sb_2_Te_3_ 2D vdW superlattices with controlled vertical thickness and lateral size. **Figure** **1**a,b presents the top‐view and side‐view scanning electron microscopy (SEM) images with theoretical schematic lattice structures of the representative Bi_2_Te_3_‐Sb_2_Te_3_ 2D vdW superlattices.

## 2 Results and Discussion

### 2.1 Material Quality Characterizations of High‐Quality 2D vdW Superlattices

Considering their potential ultimate applications in electronic devices, material quality characterization in this work mainly focuses on the structural and electronic properties of the Bi_2_Te_3_‐Sb_2_Te_3_ heterostructures/superlattices. Their structural characterization includes three main aspects: surface morphology, chemical composition, and lattice coherence.

**Figure 2:**
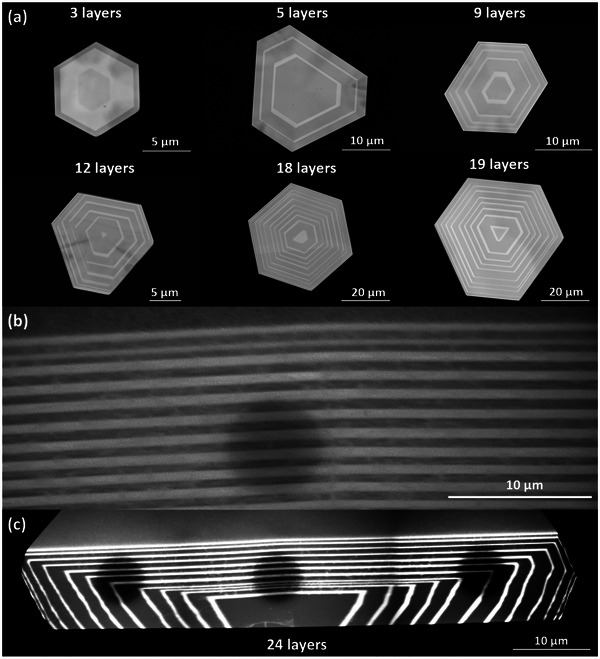
Surface morphology of 2D vdW Bi_2_Te_3_‐Sb_2_Te_3_ superlattices. a) Top‐view SEM images for Sb_2_Te_3_ (dark layers) and Bi_2_Te_3_ (bright layers) lateral superlattices when the number of layers increases from 3 to 5, 9, 12, 18, and 19; b) Side‐view SEM images for Sb_2_Te_3_ (dark layers) and Bi_2_Te_3_ (bright layers) vertical superlattices. c) Full‐scale SEM images obtained from the as‐grown wrapped superlattice with 24 layers via FIB milling/cutting.

In terms of surface morphology characterization, **Figure** **2**a presents the typical top‐view SEM images of 2D vdW lateral superlattices comprising alternating layers based on Sb_2_Te_3_ (dark layers) and Bi_2_Te_3_ (bright layers). The top‐view SEM images captured from the concentric backscattered detector in field‐free mode clearly demonstrate that, as the number of layers increases from 3 to 5, 9, 12, 18, and 19, the nanoplates maintain a hexagonal geometric shape with uniform, smooth, and flat surfaces, preserving the structural integrity of the 2D vdW superlattices. The alternating bright and dark layers show clear and sharp interfaces, indicating high‐quality CVD growth of the 2D vdW superlattices. The overall lateral size increases gradually from ≈10 to 40 µm with increasing the layer number from 3 to 19. Figure S6 in Supporting Information presents the height profile and the corresponding atomic force microscopy (AFM) images of representative 2D vdW superlattices in this work. It can be observed that the thickness of these 2D vdW superlattices can reach as small as ≈12 nm, corresponding to the thickness of a 4‐layer lattice. It should be noted that the thickness (vertical size) of these 2D vdW superlattices as well as the lateral size of each interlayer layer can be well controlled within a wide range by engineering the superlattice fabrication parameters which will be discussed in the superlattice engineering section of this work.

Figure 2b illustrates the side‐view SEM images of 2D vdW vertical superlattices comprising alternating layers based on Sb_2_Te_3_ (dark layers) and Bi_2_Te_3_ (bright layers). The side‐view SEM images were captured from the in‐column detector in immersion mode. Note that this side‐view SEM specimen was obtained from the as‐grown wrapped superlattice sample shown in Figure 2c via FIB milling/cutting. It is observed that vertical Bi_2_Te_3_‐Sb_2_Te_3_ 2D superlattices were achieved with clear and abrupt interfaces, indicating high‐quality CVD growth. Typically, the thickness (vertical size) of each layer in these 2D vdW superlattices can be well controlled within a wide range from several nanometers to several tens of nanometers by engineering the superlattice fabrication parameters. Note that the lateral size of these vertical superlattices is limited by the lateral size of the first layer of the wrapped superlattices (not wafer‐scale) which will be discussed in the superlattice engineering section later. The above results indicate our strong capability to fabricate both lateral and vertical 2D vdW superlattices especially Bi_2_Te_3_‐Sb_2_Te_3_ 2D superlattices with controlled morphology which enables engineering their physical properties and thus their device applications.

**Figure 3:**
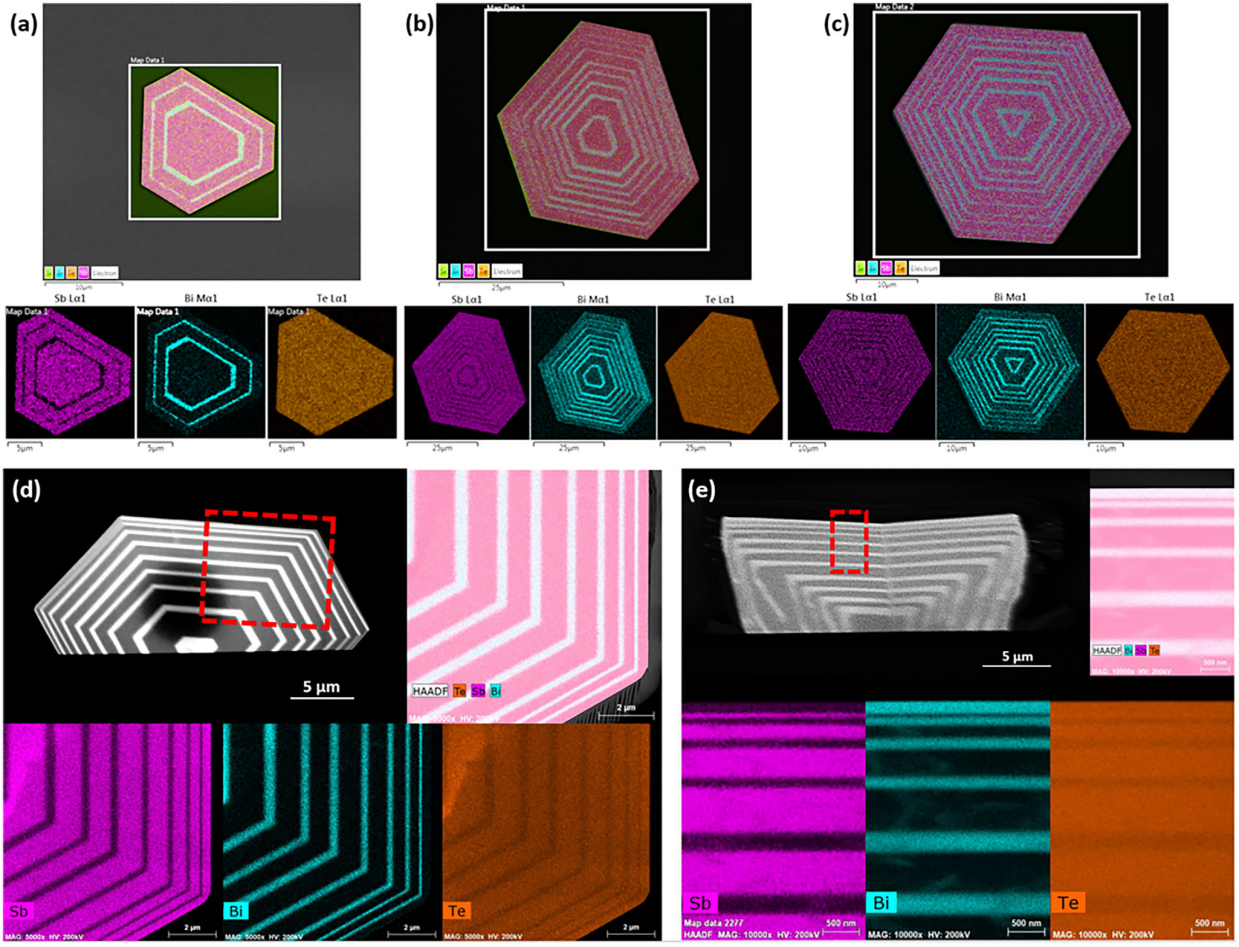
Chemical composition of 2D vdW Bi_2_Te_3_‐Sb_2_Te_3_ superlattices. Full‐scale EDX mapping of a) 5‐layer, b) 17‐layer, and c) 19‐layer lateral superlattices and corresponding elemental maps for Sb, Bi, and Te. HAADF‐STEM images and Micro‐zone EDX mapping for element Sb, Bi, and Te of d) a representative lateral 17‐layer superlattices (top‐view) and e) a representative vertical 19‐layer superlattices (side‐view). The cross‐sectional areas within the red dashed lines for both samples exhibit an alternating distribution of Bi and Sb elements, accompanied by a uniform distribution of Te elements.

In terms of chemical composition characterization, the energy‐dispersive X‐ray (EDX) spectroscopy capability of the SEM was employed to obtain full‐scale EDX mapping, which characterizes the overall elemental distribution across the lateral 2D vdW superlattices. Additionally, micro‐zone EDX mapping based on Transmission Electron Microscope (TEM) was utilized to investigate the microscopic elemental distributions in both the lateral and vertical vdW superlattices. **Figure** **3**a–c presents the full‐scale EDX mapping and the corresponding distributions of the elements Sb, Bi, and Te in Bi_2_Te_3_‐Sb_2_Te_3_ 2D vdW superlattices with 5, 17, and 19 layers, respectively. It is evident that Sb and Bi exhibit periodic alternating patterns across the different layer numbers of the 2D vdW superlattice samples. In contrast, the element Te displays a uniform distribution throughout the whole sample, reflecting its stable chemical stoichiometry in a wide area, which remains unaffected by the layer stacking. To investigate the detailed elemental distribution and chemical composition of the superlattices, the FIB cutting technique was employed to obtain cross‐sectional micro‐zone EDX images of the samples through transverse and longitudinal cutting. Figure 3d,e presents the high‐angle annular dark‐field scanning transmission electron microscopy (HAADF‐STEM) images and the micro‐zone EDX mapping of the 2D vdW superlattices in the top‐view and side‐view, respectively, along with the corresponding distributions of the elements Sb, Bi, and Te within the cross‐sectional areas indicated by the red dashed lines. The distinct boundaries between the Sb and Bi areas highlight their complementary feature in terms of chemical composition. Consistent with the full‐scale EDX mapping, the micro‐zone EDX mapping also reflects the alternating growth of Sb_2_Te_3_ and Bi_2_Te_3_, and the chemical ratio within each layer matches the expected 2:3 (Bi/Sb:Te) stoichiometric ratio, as evidenced by the elemental composition obtained from the analysis of the EDX spectra for each layer, which is shown in Figures S7, S8 in Supporting Information.

**Figure 4:**
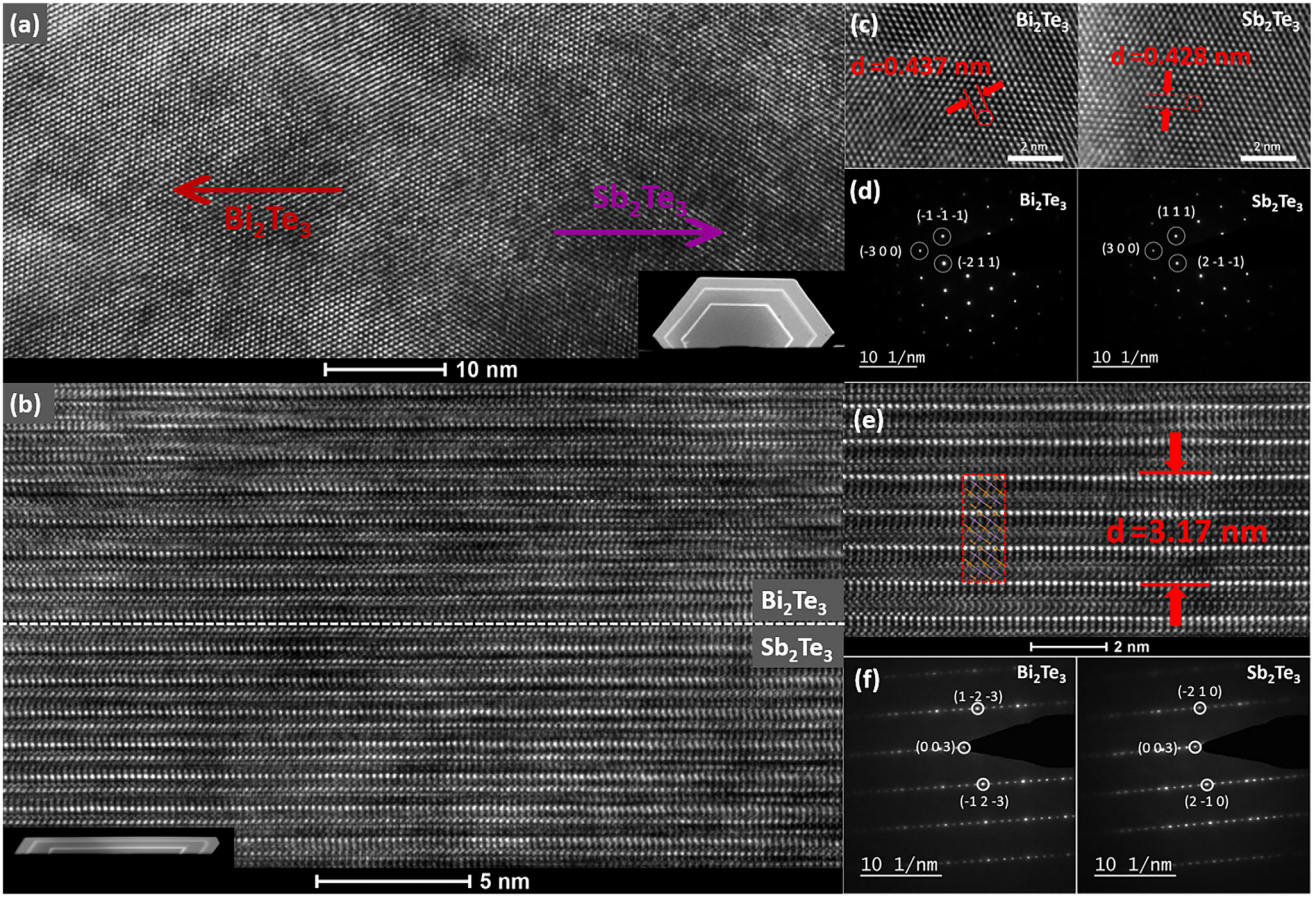
Lattice coherence of 2D vdW Bi_2_Te_3_‐Sb_2_Te_3_ superlattices. HRTEM images for the interface area of a) lateral and b) vertical superlattices obtained from the cross‐sectional region in the inset. c) HRTEM images of Bi_2_Te_3_ and Sb_2_Te_3_ lateral layers, with the measured lattice constants of ≈0.437 nm and ≈0.428 nm, respectively. d) The SAED patterns of the Bi_2_Te_3_ and Sb_2_Te_3_ layers in the lateral superlattices, respectively. e) HRTEM image of the vertical superlattices, with the measured lattice constant of ≈3.17 nm along the *c*‐axis. f) The SAED patterns of the Bi_2_Te_3_ and Sb_2_Te_3_ layers in the vertical superlattices, respectively.

In terms of lattice coherence characterization, lateral and vertical cross‐sectional TEM analyses were performed on 5‐layer 2D vdW superlattices to investigate the detailed atomic structures of the superlattices. **Figure** **4**a presents a high‐resolution TEM (HRTEM) image obtained from the cross‐sectional region in the lower‐right area of the lateral superlattices as shown in the inset. The image reveals the regular atomic arrangement at the interface between the Sb_2_Te_3_ and Bi_2_Te_3_ layers. Figure 4b shows the HRTEM image of the vertical interface between Sb_2_Te_3_ and Bi_2_Te_3_ layers, obtained from the cross‐sectional region in the lower‐left area of the wrapped superlattices as shown in the inset of Figure 4b. As shown in Figure 4b, along the vertical direction the stacked molecule structures are held by vdW forces instead of chemical bonds. The transition between these layers is remarkably smooth, without any apparent atomic mixing or defects. These indicate the high crystalline quality of the Bi_2_Te_3_‐Sb_2_Te_3_ 2D vdW superlattices grown in this work. Figure 4c shows HRTEM images of the Bi_2_Te_3_ and Sb_2_Te_3_ lateral layers of the superlattices shown in the inset of Figure 4a, respectively, at a magnification of 1.5 million times, providing a characterization of the superlattices’ crystalline quality at high spatial resolution. Both Bi_2_Te_3_ and Sb_2_Te_3_ materials constituting the superlattices exhibit highly regular atomic arrangements. The measured lattice constants of Bi_2_Te_3_ and Sb_2_Te_3_ along the *a*‐axis and *b*‐axis are ≈0.437 nm and ≈0.428 nm, respectively, which agree well with the standard database values (ICSD‐193330^[^ ^17^ ^]^ for Bi_2_Te_3_ and ICSD‐131224/ CCDC Nr. 1898614^[^ ^18^ ^]^ for Sb_2_Te_3_). Figure 4d indicates the selected area electron diffraction (SAED) patterns of the Bi_2_Te_3_ and Sb_2_Te_3_ layers in the lateral vdW superlattices shown in the inset of Figure 4a, respectively. For the Bi_2_Te_3_ lateral layer, the *d*‐spacings of the (−1 −1 −1), (−3 0 0), and (−2 1 1) crystal planes, as determined from the SAED pattern, match successfully with the standard database. For the Sb_2_Te_3_ lateral layer, the *d*‐spacings of the (1 1 1), (3 0 0), and (2 −1 −1) crystal planes, obtained from the SAED pattern, also match the standard database. Figure 4e presents an HRTEM image of the vertical stacked layers shown in the inset of Figure 4b. The measured lattice constant along the *c*‐axis is ≈3.17 nm, which matches the expected database value and aligns with the side view of the R −3 m H space group within the red dashed region. Note that since Bi_2_Te_3_ and Sb_2_Te_3_ have close lattice constants along the *c*‐axis, they are not measured separately here. Figure 4f shows the SAED patterns of the Bi_2_Te_3_ and Sb_2_Te_3_ layers in the vertical vdW superlattices shown in the inset of Figure 4b. For the Bi_2_Te_3_ vertical layer, the d‐spacings of the (1 −2 −3), (0 0 3), and (−1 2 −3) crystal planes, as determined from the SAED pattern, match successfully with the standard database. For the Sb_2_Te_3_ vertical layer, the d‐spacings of the (−2 1 0), (0 0 3), and (2 −1 0) crystal planes, obtained from the SAED pattern, also match the standard database.

**Figure 5:**
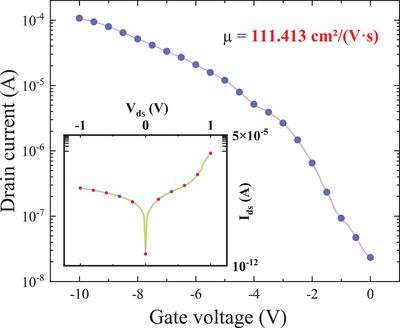
Room‐temperature transfer characteristic for the FET with 1 V applied bias voltage, indicating n‐type transport behavior. Inset: I_ds_–V_ds_ curve acquired in a dark environment.

Apart from the high structural quality discussed above, the Bi_2_Te_3_‐Sb_2_Te_3_ 2D superlattices also show excellent electronic properties benefiting their ultimate device applications. To evaluate their electronic properties, back‐gated field‐effect transistors (FET) were fabricated, and their source‐drain current versus gate voltage (I_ds_−V_g_) characteristics were analyzed to extract information about electron mobility within. The device schematic was included in Figure S12a of Supporting Information. The transfer characteristics of the fabricated superlattice FET, obtained with a gate voltage (V_g_) under dark conditions, are presented in **Figure** **5**. With the I_ds_, V_ds,_ and FET device dimensions, the carrier mobility μ can be extracted to be 111.41 cm^2^ V^−1^ s^−1^ at room temperature by using the relationship between carrier mobility μ and the parameters (I_ds_, V_ds_ and FET device dimension),^[^ ^19^ ^]^ the details of which can be found in the Supporting Information. This electron mobility (111.41 cm^2^ V^−1^ s^−1^) extracted is comparable to those reported for most high‐quality single 2D materials, including MoS_2_ (0.5–200 cm^2^ V^−1^ s^−1^),^[^ ^19b^ ^]^ WS_2_ (50 cm^2^ V^−1^ s^−1^),^[^ ^20^ ^]^ and WSe_2_ (250 cm^2^ V^−1^ s^−1^).^[^ ^21^ ^]^ In addition, the I_ds_–V_ds_ characteristics (insert of Figure 5) exhibit clear nonlinear behavior with significant current flow in the positive voltage region while showing much lower current in the negative voltage region. This asymmetric transport behavior is the typical rectifying characteristic of a p‐n junction, which was observed in previous work.^[^ ^22^ ^]^

Note that the FET device exhibits an exceptionally high on‐off ratio of 5818. Considering the small difference between the bandgaps of these two materials, it can be explained through the differences in their interfacial band structures. Specifically, we calculated the electron affinity(*χ*)and ionization energy(*I*)for both materials: For Bi_2_Te_3_, considering its bandgap *E*_*g*1_ =  0.15 *eV*, electron affinity χ_1_ =  5.3 *eV*, and consequently, its ionization energy  *I*_1_ = χ_1_  +  *E*_*g*1_ =  5.45 *eV*; similarly for Sb₂Te₃, we determined its corresponding parameters. For Sb_2_Te_3_, it's bandgap *E*_*g*2_ =  0.28 *eV*, and electron affinity χ_2_ =  5.0 *eV*, consequently, its ionization energy *I*_2_ = χ_2_  +  *E*_*g*2_ =  5.28 *e* *V*. Utilizing these parameters, we established the band offsets at the interface. The valence band offset (Δ *E_v_* = *I*_1_  −  *I*_2_ =  0.17 *eV*), indicates that the valence band maximum of Sb₂Te₃ is 0.17 eV lower than that of Bi₂Te₃, while the conduction band offset reveals that the conduction band minimum of Bi₂Te₃ is 0.3 eV (Δ *E_c_* = χ_1_  −  χ_2_ =  0.3 *eV*) higher than that of Sb₂Te₃. This Type‐II band alignment creates an electron barrier and a hole well at the interface. By applying an electric field *E* across the oxide insulating layer, the barrier height *Φ* (where *q* is the elementary charge and *d* is the barrier width) can be modulated, significantly affecting the carrier tunneling probability.^[^ ^23^ ^]^

According to the thermionic emission theory, the current density *J* exhibits an exponential dependence on the barrier height *Φ*, expressed as:

(1) $$J=A\cdot T^{2}exp\left(-\frac{\Phi }{kT}\right)$$

where *A* is the Richardson constant, *k* is the Boltzmann constant, and *T* is the absolute temperature.^[^ ^24^ ^]^ This relationship demonstrates that current density is highly sensitive to changes in barrier height. When an electric field *E* is applied across the oxide insulating layer, the interfacial barrier height modulation can be expressed as:

(2) $$\Delta \Phi =\frac{Edq}{2}$$

where *q* is the electronic charge and *d* is the barrier width.^[^ ^25^ ^]^ Through the electrical field modulation, we can precisely control the barrier height *Φ*. Even small changes in barrier height *ΔΦ* lead to substantial changes in current density *J* due to the exponential relationship, explaining the high on/off ratio observed in our experiments: in the “on” state, the electric field reduces the barrier height, significantly increasing the current; in the “off” state, the electrical field increases the barrier height, substantially reducing the current.

Moreover, as topological insulators, both Bi₂Te₃ and Sb₂Te₃ possess unique topological surface states. These states exhibit linear dispersion near the Fermi level and are protected by time‐reversal symmetry, resulting in high mobility and resistance to scattering.^[^ ^26^ ^]^ The application of electric field *E* influences the properties of topological surface states through several mechanisms: 1) Energy Level Modulation: The electric field induces band bending, altering the relative position of surface states with respect to the Fermi level, thereby affecting surface state occupation and carrier concentration.^[^ ^27^ ^]^ 2) Mass Gap Introduction: Strong electric fields may break time‐reversal symmetry, introducing a mass gap in the surface states and modifying their conductivity.^[^ ^28^ ^]^ 3) Scattering Mechanism Modification: The electrical field can enhance or suppress scattering between surface and bulk states, affecting overall electronic transport properties.^[^ ^29^ ^]^ This dual modulation mechanism allows device conductivity to vary over a broader range, explaining the significant switching behavior we observed. Note that this enhancement of the electrical properties in topological insulators has been previously reported in the literature.^[^ ^30^ ^]^

It is interesting to observe that the Bi_2_Te_3_‐Sb_2_Te_3_ superlattices demonstrate p‐type transistor characteristics despite involving both n‐type Bi_2_Te_3_ and p‐type Sb_2_Te_3_ materials. This observation can be mainly explained by the following mechanisms. As shown in Figure S12b (Supporting Information), the band alignment between Bi_2_Te_3_ and Sb_2_Te_3_ leads to a Type‐II heterostructure with a valence band offset (Δ*E_v_*) of 0.17 eV and conduction band offset (Δ*E_c_*) of 0.3 eV. The key concept is the “energetic barrier” – carriers need to overcome energy differences to move between materials. Since the valence band offset (0.17 eV) is smaller in magnitude than the conduction band offset (0.3 eV), holes face a smaller energy barrier than electrons when crossing between these materials. So, in such a band configuration, hole transport is more favorable than electron transport because of the lower energetic barrier in the valence band.^[^ ^31^ ^]^ In addition, for thin Bi_2_Te_3_/Sb_2_Te_3_ structures p‐type transport can dominate due to the topological surface states as reported previously.^[^ ^32^ ^]^

These results provide a clear characterization of our Bi₂Te₃‐Sb₂Te₃ superlattices, confirming their high crystal quality and electronic properties within.

### 2.2 CVD Growth Mechanism and Kinetics of 2D vdW Superlattices

Based on the extensive characterization results of the 2D vdW superlattice as shown above, we proposed a novel growth model, using the example of Bi_2_Te_3_‐Sb_2_Te_3_ to elucidate the growth mechanism of 2D vdW superlattices based on V–VI binary chalcogenides in this work.

**Figure 6:**
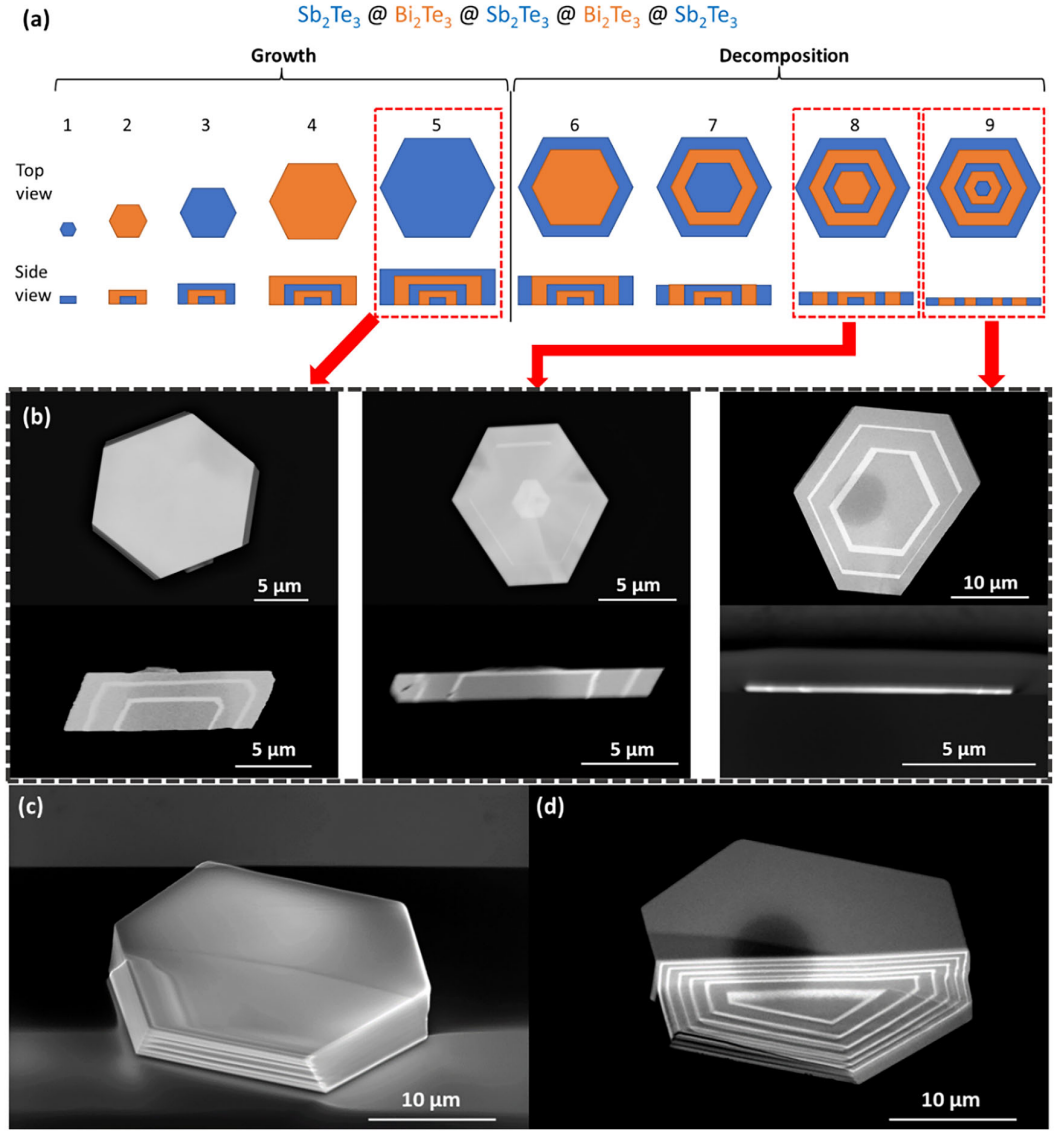
Schematic CVD growth model of 2D vdW Bi_2_Te_3_‐Sb_2_Te_3_ superlattices: a) Growth sequence of 5‐layer superlattices from the top‐view and side‐view perspectives, exemplified by Bi_2_Te_3_‐Sb_2_Te_3_. The completed synthesis process is divided into two main parts: growth and decomposition, consisting of a total of nine steps. b) SEM images of representative 2D vdW superlattice structures in Step 5, Step 8, and Step 9 from both the top‐view and side‐view perspectives. c) SEM image of a representative 13‐layer superlattice sample, and d) its cross‐sectional SEM image transected with FIB milling.

As shown in **Figure** **6**a, the fabrication process of a 5‐layer Bi_2_Te_3_‐Sb_2_Te_3_ 2D vdW superlattices can be separated into two primary stages: growth and decomposition, consisting of nine steps. In the first step, Sb_2_Te_3_, acting as precursor A, is initially pushed into the central heating zone of the tubular furnace, and subsequently nucleates on the substrate surface. Bi_2_Te_3_, as precursor B, then alternates with Sb_2_Te_3_ for four growth cycles, with each precursor material continuing the epitaxy on the outer surface of the existing crystal, resulting in the wrapped superlattice structures shown in steps 2–5. Note that, in contrast to most previously reported growth models for 2D heterostructures/superlattices,^[^ ^33^ ^]^ the precursor materials transported by the carrier gas to the substrate surface exhibit a wrapping‐style epitaxy on the existing nanostructures, rather than in‐layer lateral epitaxy. Therefore, at the end of step 5, the obtained superlattices are usually thick samples with a layered structure alternately wrapped by different precursor materials, as shown in Figure 6b. These thick superlattices are held together by vdW forces between the layers, while the in‐layer atoms are interconnected by chemical bonds. To further confirm the formation of these wrapped superlattices, FIB milling/cutting was employed to transect these wrapped superlattices. Figure 6c shows a representative SEM image of 13‐layer superlattices corresponding to the growth sequence at step 5, while Figure 6d shows the related cross‐sectional SEM image transected with FIB milling. The wrapped superlattices are clearly observed in the 13‐layer wrapped superlattices.

**Figure 7:**
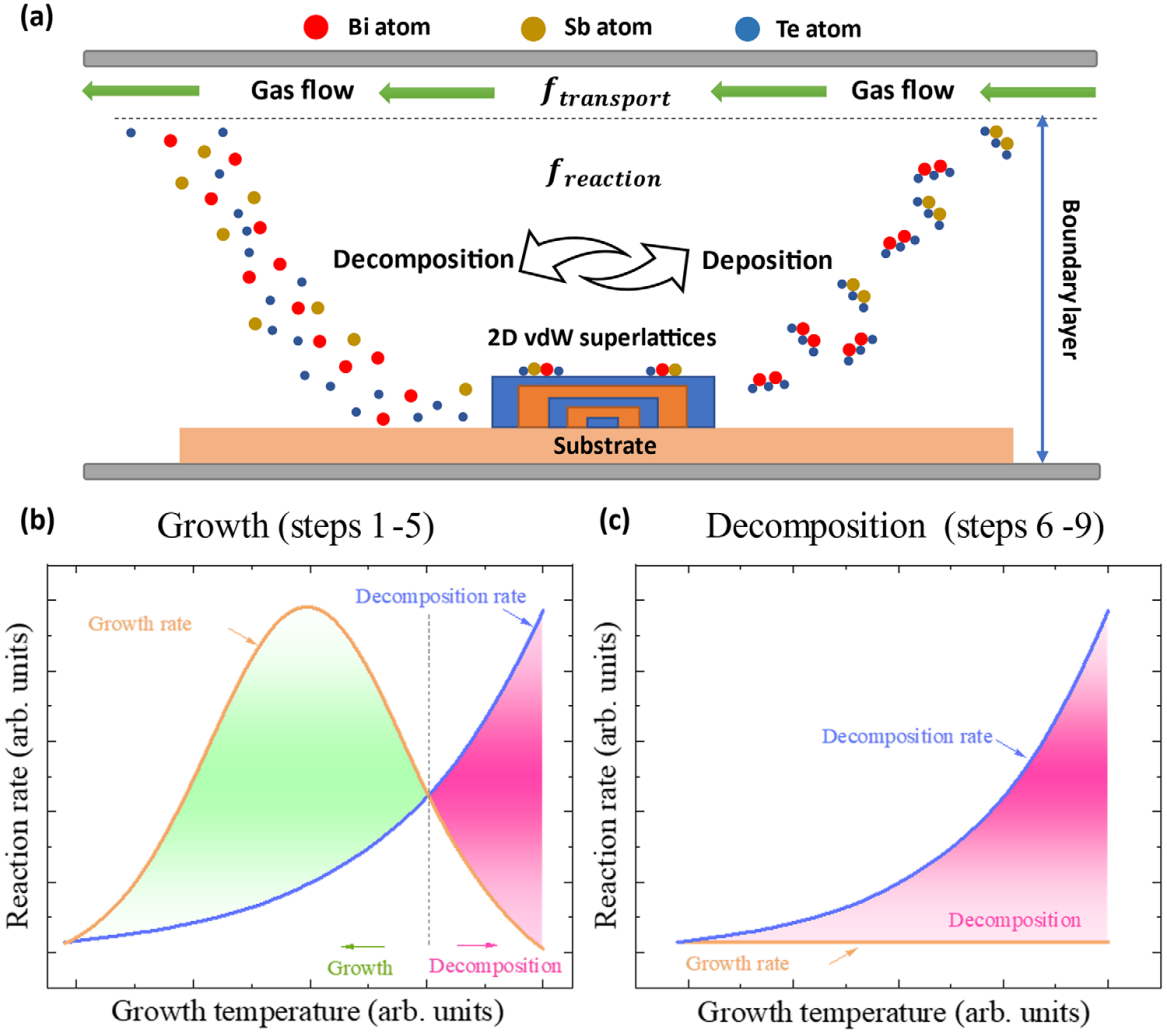
CVD Growth mechanism for the 2D vdW superlattices in this work. a) Schematic growth model for phase deposition synthesis of 2D vdW superlattices, exemplified by Bi_2_Te_3_‐Sb_2_Te_3_. Note that the boundary layer is assumed stagnant because of the steady gas flow. Growth temperature‐dependent reaction rate for b) Growth part and c) Decomposition part. In the decomposition part, the growth rate is constant at 0 due to the absence of carrier gas flow.

To elucidate a comprehensive explanation for the CVD growth mechanism of the 2D vdW superlattices in this work, we introduce a classic CVD growth model to analyze the detailed reactions occurring on the substrate surface,^[^ ^34^ ^]^ as shown in **Figure** **7**a. Upon initiation of the reaction, the needle valve of the CVD growth system is opened, allowing the precursor molecules to be carried by the carrier gas flow to the substrate surface. Owing to the relatively low temperature of the substrate surface, some molecules pass through the boundary layer and deposit on the substrate. Subsequently, the precursor molecules undergo decomposition, forming active atoms, which diffuse along the substrate surface and nucleate at preferential energy sites, followed by continuous epitaxial growth. Concurrently, the decomposition of the crystal also takes place, maintaining a dynamic balance between the growth and decomposition processes. The decomposed atoms, along with unreacted atoms, desorb from the substrate surface, diffuse through the boundary layer, and are ultimately carried away by the carrier gas flow. Generally, in traditional CVD growth processes, crystal growth and decomposition exist simultaneously on the substrate surface.^[^ ^35^ ^]^ In practical situations, the rates of epitaxial growth and decomposition could differ owing to minor temperature variances on the substrate surface. The temperature of the substrate surface at which crystals grow is defined as growth temperature. Growth temperature plays a crucial role in the nucleation and epitaxy of the nanostructures, ultimately influencing their shape, size, distribution, and others. Figure S9 in Supporting Information shows the growth temperature distribution on the substrate surface obtained via a thermodynamic analysis of the growth process within the tube furnace.

Theoretically, in regions where the growth rate exceeds the decomposition rate, the superlattice crystal maintains a growth tendency, i.e., the green area in Figure 7b; while in regions where the decomposition rate exceeds the growth rate, superlattices with fewer layers in that region decompose, as illustrated in the pink area in Figure 7b,c. In the CVD process of this work, when the experimental procedure reaches the final step, the precursor materials are extracted, and the heating process terminates. At this stage, on the substrate surface, the influx of precursor molecules transported by the carrier gas is zero as the precursor materials are extracted, leading to a zero crystal growth rate and thus inhibiting the further growth of superlattice crystals. However, the decomposition process continues due to the relatively high temperature of the sample surface because of the slow cooling process, as shown in Figure 7c. Note that similar growth and decomposition curves have been reported in other CVD studies.^[^ ^36^ ^]^ This decomposition process continues until the temperature of the sample surface drops below the minimum temperature required for sustaining crystal decomposition. Due to the weaker vdW forces in comparison to the chemical bonds, the thick 2D vdW superlattices decompose layer‐by‐layer along the vertical dimension (*c*‐axis), as depicted in Figure 6a (steps 6–9). In contrast, benefiting from the strong chemical bonds the atomic structures along the lateral dimension remain relatively intact, leading to the formation of lateral 2D vdW superlattices. Therefore, lateral superlattices with various vertical thicknesses can be achieved by tuning the furnace cooling rate.

The decomposition rate of superlattices is primarily driven by thermodynamic instability and diffusion processes. As the surface temperature remains relatively high, atoms at the interfaces between layers have sufficient energy to overcome diffusion barriers. The kinetics of decomposition can be described by an Arrhenius‐type equation:

(3) $$R_{d}=a\cdot exp\left(-\frac{E_{a}}{kt}\right)$$

where *R_d_* represents the decomposition rate, *a* is the pre‐exponential factor, *E_a_* is the activation energy, and *k* is the Boltzmann constant.^[^ ^37^ ^]^ A higher cooling rate reduces the time available for atomic diffusion, thereby preserving the superlattice structure more effectively, and leading to better crystal quality for the resultant superlattices. However, cooling too fast can cause damage to the CVD system. In this work, a cooling rate of 25 °C min^−1^ is used which results in high‐quality superlattices.

Due to the random nature of precursor nucleation, each precursor switch during the 2D superlattice growth could result in new nucleation sites on the substrate surface, leading to the formation of 2D vdW superlattices with different numbers of layers. This explains the observation of 4‐layer and 3‐layer superlattices in the 5‐layer superlattice growth experiment. It also should be noted that the growth of 2D single Bi_2_Te_3_ and Sb_2_Te_3_ materials are highly anisotropic^[^ ^38^ ^]^: a large growth rate along the lateral direction, while a very small growth rate along the vertical direction. Thus, the thickness of the individual layers of the wrapped superlattice is very small as evidenced by the cross‐sectional TEM results in the previous discussion. CVD growth parameters such as a higher precursor temperature, longer growth time, higher carrier gas flow rate, lower inner tube pressure, and others can lead to a larger lateral growth rate and thus a larger lateral size for the individual layers of the wrapped superlattices.^[^ ^39^ ^]^ Despite having similar changing trends their impact on the vertical growth rate and thickness is much smaller in comparison to that in the lateral growth rate and size. It is interesting to note that all superlattices grown in this work present excellent uniformity in terms of thickness as well as interface sharpness and flatness regardless of the changes in CVD growth parameters, which is evidenced by the SEM and TEM results in the previous discussion. It should also be noted that despite the change of CVD growth parameters including Argon carrier gas flow rate the 2D nanostructures, including both single layer and heterostructures/superlattices present high materials quality once the CVD growth parameters are appropriate to form these nanostructures.

### 2.3 Superlattice Engineering

As discussed before, the as‐grown Bi_2_Te_3_‐Sb_2_Te_3_ 2D vdW superlattices are wrapped superlattices, and the thickness and lateral size of each interlayer can be well controlled by tuning the CVD growth parameters such as precursor temperature, carrier gas flow rate, growth time, and tube inner pressure. Figure S10 in the Supporting Information shows the most complex wrapped Bi_2_Te_3_‐Sb_2_Te_3_ 2D vdW superlattices grown in our lab with 32 layers, indicating our strong capability in growing Bi_2_Te_3_‐Sb_2_Te_3_ 2D vdW superlattices. These wrapped superlattices might present intriguing physical properties due to the special wrapping shape for each constituting layer. However, for practical industry applications, it is preferred to have either lateral superlattices or traditional vertical superlattices which are more feasible for device fabrication. It should be noted that the current industrial process for device fabrication is mainly based on vertical heterostructures/superlattices grown with epitaxial techniques such as molecular beam epitaxy (MBE) and metal‐organic chemical vapor deposition (MOCVD).^[^ ^40^ ^]^ Therefore, it is essential to engineer the wrapped superlattices to achieve either lateral or vertical superlattices, especially vertical ones.

**Figure 8:**
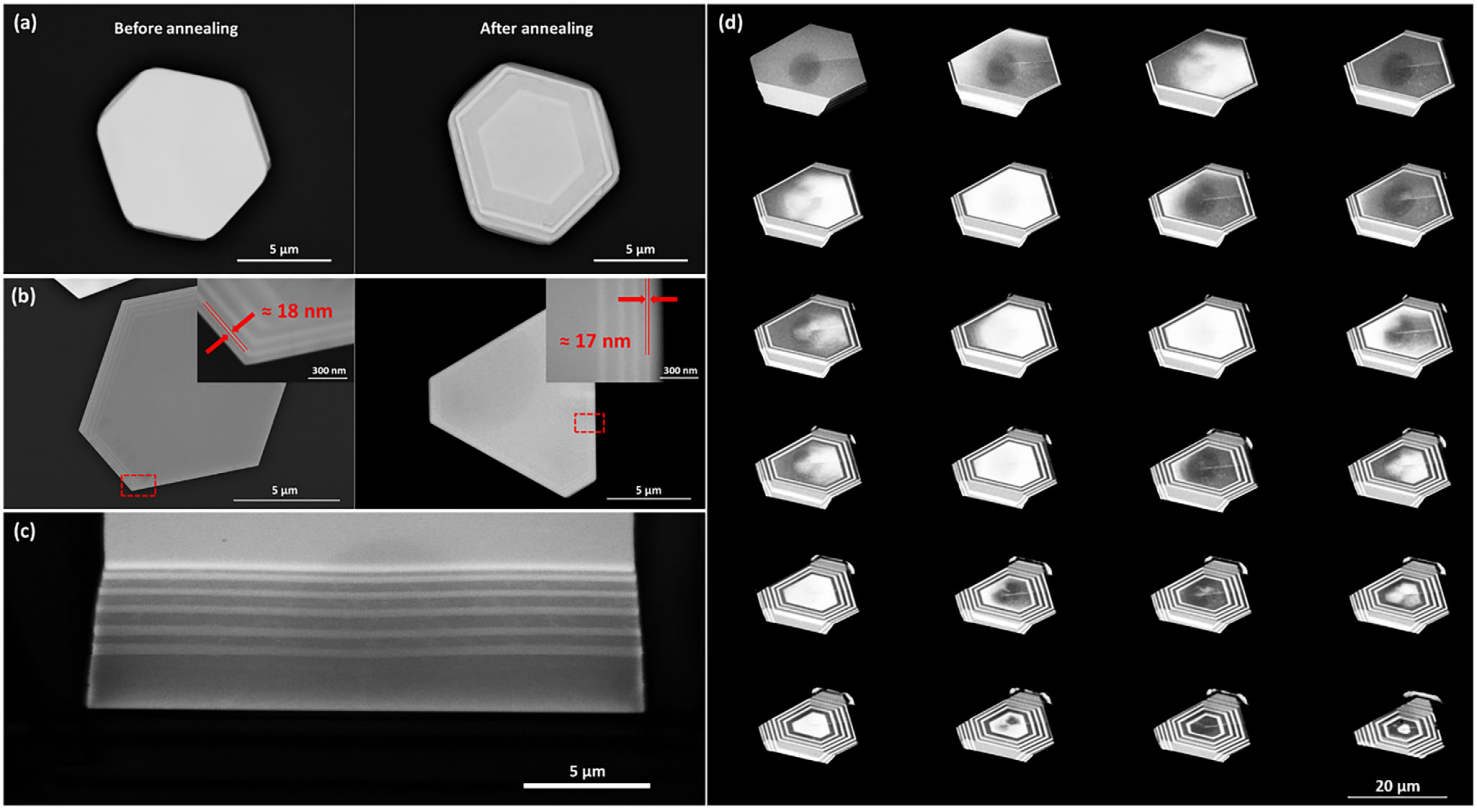
Superlattice Engineering. a) SEM images of 2D vdW Bi_2_Te_3_‐Sb_2_Te_3_ superlattice before and after annealing under vacuum at 545 °C for 60 min. b) SEM images of representative 9‐layer Bi_2_Te_3_‐Sb_2_Te_3_ samples with a small lateral width (≈17 and 18 nm) were obtained with a small growth time of 2 min. c) SEM images for Bi_2_Te_3_‐Sb_2_Te_3_ vertical superlattices obtained with FIB cutting. d) In situ SEM images show the whole FIB milling process to peel off the 15‐layer Bi_2_Te_3_‐Sb_2_Te_3_ wrapped superlattices layer by layer to achieve lateral superlattices.

The vertical thickness of the lateral superlattices can be controlled by applying the annealing process and tuning the annealing time. Being analogous to the sample's natural cooling process, post‐growth annealing under vacuum conditions is proposed in this work as a more controllable approach to decompose the wrapped superlattices. **Figure** **8**a shows the Bi_2_Te_3_‐Sb_2_Te_3_ lateral superlattices obtained before and after annealing. The Bi_2_Te_3_‐Sb_2_Te_3_ wrapped superlattices were obtained under vacuum at 545 °C for 60 mins. Clearly, Bi_2_Te_3_‐Sb_2_Te_3_ lateral superlattices are achieved with their vertical thickness controlled by the annealing temperature and annealing time.

The width for each layer of the lateral superlattices can be controlled by tuning the growth time. Figure 8b shows the top‐view SEM image of representative Bi_2_Te_3_‐Sb_2_Te_3_ lateral superlattices obtained in this work. It is clearly observed that the width of each layer in the superlattices can be controlled to be within the quantum confinement regions (<20 nm), constituting a true superlattice structure.

In contrast to lateral 2D vdW superlattices, vertical 2D vdW superlattices are preferred as they are more compatible with the current advanced device fabrication process. For example, the current semiconductor lasers and photodetectors are mainly based on mesa structures which are vertical heterostructures or superlattices.^[^ ^41^ ^]^ Then, how to achieve vertical superlattices from the as‐grown wrapped superlattices? Here, FIB cutting is proposed as a damage‐free technique to cut the as‐grown wrapped superlattices and achieve vertical superlattices. Note that FIB cutting is used widely for preparing TEM specimens without damage, which allows cutting the wrapped superlattices without damage. Figure 8c shows the Bi_2_Te_3_‐Sb_2_Te_3_ vertical superlattices obtained with FIB cutting. Clearly, Bi_2_Te_3_‐Sb_2_Te_3_ vertical superlattices with clear and abrupt interfaces are obtained, indicating the high structural quality of the vertical superlattices obtained and the feasibility of this FIB cutting for achieving vertical superlattices. Furthermore, FIB milling can also be used for achieving lateral superlattices. The wrapped superlattices can be peeled off layer‐by‐layer from the top surface with small‐current FIB milling to achieve lateral superlattices. Figure 8d shows the SEM images of the whole FIB milling process for achieving Bi_2_Te_3_‐Sb_2_Te_3_ lateral superlattices. Clearly, FIB milling/cutting provides a powerful approach for engineering the wrapped superlattices to achieve both lateral and vertical superlattices. It should be noted that the FIB milling/cutting process is time‐consuming, and thus not suitable for large‐scale industry manufacturing. Therefore, alternative industry fabrication processes should be studied in the future to meet the industry requirement of scalability and uniformity such as reactive ion etching and nanoimprint lithography.

Additionally, to validate the applicability of the above 2D superlattice growth model to other V–VI binary chalcogenides, we further investigated alternative material combinations, e.g., Bi_2_Se_3_/Sb_2_Te_3_. Figure S11 in Supporting Information shows the full‐scale EDX mapping of the 2‐layer lateral 2D vdW heterostructure and the corresponding elemental distributions for Bi, Se, Sb, and Te. It is observed that Bi_2_Se_3_/Sb_2_Te_3_ heterostructures are achieved with clear interfaces and uniform elemental distributions. This suggests that the 2D vdW superlattice growth model proposed in this work shows general applicability across the family of chalcogenide materials.

In all, CVD growth provides a low‐cost approach for achieving high‐quality 2D vdW heterostructures/superlattices through its material quality, and thus device performance might not be as super as those grown with MBE and MOCVD. This will enable it to be feasible to fabricate electronic devices for various commercial applications.

## 3 Conclusion

In this work, we report a comprehensive study on the superlattice engineering of 2D chalcogenides. Theoretical DFT calculations suggested the feasibility and stability of Bi_2_Te_3_‐Sb_2_Te_3_ 2D vdW superlattices due to their low interface energy. With an innovative precursor switching approach, 2D vdW Bi_2_Te_3_‐Sb_2_Te_3_ wrapped superlattices were achieved by CVD growth. With superlattice engineering approaches such as thermal decomposition and FIB milling/cutting, both lateral and vertical Bi_2_Te_3_‐Sb_2_Te_3_ 2D vdW superlattices were achieved with clear and sharp interfaces between constituting layers. Further characterization analysis on both the structural properties and the electronic properties revealed the high material quality of the 2D vdW superlattices. A growth model was introduced to understand the growth mechanism and fabrication process of these superlattices. This interesting and innovative study provides a feasible approach to fabricate three different types of superlattices: wrapped, lateral, and vertical superlattices, and thus lays the foundation for further studying their device applications.

## 4 Experimental Section

### DFT Calculations of 2D vdW Superlattices

The first‐principles calculations based on the DFT via Materials Studio software were performed to explore the stability and experimental feasibility of the layered 2D vdW superlattice based on Bi_2_Te_3_‐Sb_2_Te_3_. The detailed DFT calculation procedures are described in Supporting Information. Figure S1a–d (Supporting Information) displays the schematic Sb_2_Te_3_ and Bi_2_Te_3_ bulk crystal lattice structures in top‐view and side‐view, respectively. Sb_2_Te_3_ and Bi_2_Te_3_ have similar rhombohedral tetradymite crystal structures, both falling within the space group R‐3 m H.^[^ ^42^ ^]^ They are layered materials, comprising multiple planar Te‐Sb (Bi)‐Te‐Sb (Bi)‐Te quintuple layers connected by vdW interactions along the *c*‐axis.^[^ ^43^ ^]^ Figure S1e,f (Supporting Information) exhibit the schematic lattice diagrams of optimized Bi_2_Te_3_‐Sb_2_Te_3_ vertical heterostructure (two layers) and superlattice (three layers), respectively. The Bi_2_Te_3_‐Sb_2_Te_3_ vertical heterostructure comprises a unit cell of quintuple‐layer (QL) Sb_2_Te_3_ and a unit cell of QL Bi_2_Te_3_ with an interlayer distance of 3.328 Å along the *c*‐axis. The Bi_2_Te_3_‐Sb_2_Te_3_ vertical superlattice consists of two unit‐cells of QL Sb_2_Te_3_ with one unit‐cell of QL Bi_2_Te_3_ sandwiched in between, and the interlayer distances are 3.294 and 3.300 Å along the *c*‐axis. Figure S1g,h (Supporting Information) indicates the schematic lattice diagrams of optimized lateral heterostructure (two layers) and superlattice (three layers) of Bi_2_Te_3_‐Sb_2_Te_3_, respectively. The Bi_2_Te_3_‐Sb_2_Te_3_ lateral heterostructure is composed of a 3 × 1 unit‐cell of QL Sb_2_Te_3_ and a 3 × 1 unit‐cell of QL Bi_2_Te_3_ connected by intra‐planar covalent bonding; while Bi_2_Te_3_‐Sb_2_Te_3_ lateral superlattice contains one additional 3 × 1 unit cell of QL Sb_2_Te_3_ compared to the lateral heterostructure, which is covalently bonded to the QL Bi_2_Te_3_ on the other side.

To evaluate the thermodynamic stability of Bi_2_Te_3_‐Sb_2_Te_3_ vertical and lateral heterostructure/superlattice, the interface forming energies were calculated as detailed in Supporting Information. The stability of heterostructure/superlattice is correlated with the magnitude of its interface energy: the lower the interface energy, the greater the stability of the structure.^[^ ^44^ ^]^ The calculated interface forming energies of Bi_2_Te_3_‐Sb_2_Te_3_ heterostructure/superlattice can be observed in Table S1 (Supporting Information). These values are positive and smaller than previously reported data of corresponding vertical and lateral heterostructures,^[^ ^45^ ^]^ indicating the high feasibility of growing Bi_2_Te_3_‐Sb_2_Te_3_ heterostructure/superlattice with high stability. Furthermore, the low interface energy calculated and thus the high stability of these heterostructures/superlattices can potentially translate into various advantages including 1) robust device fabrication process and high device yield, 2) robust operation and long lifetime, and 3) high interface quality and high device performance. This can lead to various device applications including high‐performance photodetector and field effect transistor devices, as well as thermoelectric devices.^[^ ^46^ ^]^

### Controlled CVD Growth Process of 2D vdW Superlattices

The 2D vdW superlattice synthesis utilized a novel multi‐compartment CVD setup with precursors in separate quartz boats, controlled by external magnets. Silicon wafers with 300 nm oxide served as substrates. The process involved alternating growth steps: First, precursor A was heated to its melting point and transported via argon gas to the substrate for epitaxial growth. After a set time, this was paused, and the temperature B was adjusted for precursor B, which was then similarly deposited onto crystal A. By repeating these alternating steps, multilayer structures were achieved. Finally, the samples were removed after the tubular furnace had cooled naturally down to room temperature. Refer to Table S11 in Supporting Information for detailed growth parameters.

## Conflict of Interest

The authors declare no conflict of interest.

## Supporting information



Supporting Information

## Data Availability

The data that support the findings of this study are available from the corresponding author upon reasonable request.
