# Supplementary material for: Superlattice Engineering on 2D Bi2Te3‐Sb2Te3 Chalcogenides
Source: Adv Sci (Weinh). 2025 Apr 7;12(26):2503492. doi: 10.1002/advs.202503492 (PMC12245118; doi:10.1002/advs.202503492)
Supplement: Supplementary file 1 — Supporting Information [file ADVS-12-2503492-s001.docx]

Supporting Information

**Superlattice Engineering on 2D Bi_2_Te_3_-Sb_2_Te_3_** **Chalcogenides**

*Han Wang^1, a^, Songqing Zhang^1, a^, Huijia Luo^1^, Wenwu Pan^1^, Zekai Zhang^1^, Junliang Liu ^2, ^[[1]](#footnote-2)^*)^, Yongling Ren^1^, Cailei Yuan^3^, and Wen Lei^1, ^[[2]](#footnote-3)^*)^*

^1^ Department of Electrical, Electronic and Computer Engineering, The University of Western Australia, Crawley, WA 6009, Australia.

^2^ School of Integrated Circuits, Jiangnan University, Wuxi 214122, China.

^3^ Jiangxi Key Laboratory of Nanomaterials and Sensors, School of Physics, Communication and Electronics, Jiangxi Normal University, 99 Ziyang Avenue, Nanchang 330022, China.

Contents

[Equipment 2](#_Toc193582385)

[DFT Calculation 2](#_Toc193582386)

[(1) Structure Constructions 3](#_Toc193582387)

[(2) Interface Energy Calculations 12](#_Toc193582388)

[Characterization 14](#_Toc193582389)

[Operational Procedures, Scalability and Uniformity of FIB Technique 16](#_Toc193582390)

[Device Fabrication and Characterization 17](#_Toc193582391)

[Reference 19](#_Toc193582392)

## Equipment

The 2D van der Waals (vdW) superlattices presented in this paper are obtained by reacting Sb_2_Te_3_ and Bi_2_Te_3_ powder (purity 99.99% Sigma-Aldrich) placed in a 24-inch dual-zone horizontal tube furnace (Lindberg/Blue M). The surface morphology of the 2D vdW superlattices was characterized by an atomic force microscope (AFM, Witec alpha 300RA^+^) and a scanning electron microscope (SEM, FEI Verios 460L). The crystalline structure of the 2D vdW superlattices was studied using a high-resolution transmission electron microscope (HRTEM, FEI Titan G2 80-200 TEM/STEM) equipped with selected-area electron diffraction (SAED). The chemical composition of the 2D vdW superlattices was analyzed with Energy-dispersive X-ray (EDX) spectroscopy (Oxford Instruments X-Max 80 EDS system) and the high-angle annular dark-field scanning transmission electron microscopy (HAADF-STEM, FEI Titan G2 80-200 TEM/STEM). The electronic properties of the 2D vdW superlattices device were characterized in a probe station (Lakeshore TTP4) with a semiconductor parameter analyzer (Keithley 4200 SCS).

## DFT Calculation

The Cambridge Sequential Total Energy Package (CASTEP) module in Materials Studio was utilized for the first-principles calculations of Bi_2_Te_3_-Sb_2_Te_3_ vertical and lateral heterostructures/superlattices based on DFT. The exchange-correlation effects were determined by the Perdew Burke Ernzerhof (PBE) functional within the generalized gradient approximation (GGA), and the electron-ion interactions were modulated by the generated on the fly (OTFG) norm conserving pseudopotential. According to the convergence test, the cutoff energy for Bi_2_Te_3_-Sb_2_Te_3_ vertical and lateral heterostructures/superlattices were decided to be 400 eV and 440 eV, respectively. The tested Monkhorst-Pack k-point meshes were set as 6 × 6 × 1 and 1 × 6 × 1, in which the former is for Bi_2_Te_3_-Sb_2_Te_3_ vertical heterostructures/superlattices and the latter is for lateral heterostructures/superlattices. The nonbonding vdW interactions between the quintuple-layer of Sb_2_Te_3_ and Bi_2_Te_3_ were described by DFT-D of vdW dispersion corrections. A vacuum slab of 15 Å was set to prevent the interactions between adjacent cells. All structures were optimized until the energy converged to 1 × 10^–6^ eV atom^−1^ for static total energy calculations.

**Table S1.** Calculated interface forming energies of Bi_2_Te_3_-Sb_2_Te_3_ heterostructures/superlattices.

| **Structures** | ***ΔE* (meV Å^-2^)** |
| --- | --- |
| Bi_2_Te_3_-Sb_2_Te_3_ vertical heterostructure | 1.560 |
| Bi_2_Te_3_-Sb_2_Te_3_ vertical superlattice | 0.811 |
| Bi_2_Te_3_-Sb_2_Te_3_ lateral heterostructure | 39.863 |
| Bi_2_Te_3_-Sb_2_Te_3_ lateral superlattice | 79.285 |

### (1) Structure Constructions

The configuration processes of Bi_2_Te_3_-Sb_2_Te_3_ vertical and lateral heterostructures/superlattices involved several main steps:

1. ***Bulk structure construction and optimization***

Initially, bulk structures of Sb_2_Te_3_ and Bi_2_Te_3_ were configured, and the convergence tests were conducted to determine the optimal cutoff energy and k-point mesh values, as shown in **Table S2-S3**. Our convergence criterion was based on the change in single-point energy per atom between two adjacent calculations with increasing cutoff energy or k-point mesh. We considered the parameters converged when this energy difference fell within 0.1 to 2 meV/atom, ensuring a balance between accuracy and computational efficiency. Based on these tests, 320 eV and 440 eV were selected as the optimal cutoff energies for bulk Sb_2_Te_3_ and Bi_2_Te_3_, respectively. For k-point mesh, we used Monkhorst-Pack grids of 4 × 4 × 1 for both bulk Sb_2_Te_3_ and Bi_2_Te_3_. Note that the k-point mesh is conventionally set to be 1/a:1/b:1/c.

**Table S2.** Convergence tests for bulk Sb_2_Te_3_.

| Cutoff energy (eV) | k-point mesh | Single-point energy (eV) | Difference (meV/atom) |
| --- | --- | --- | --- |
| 280 | 4×4×1 | -7262.393832 | 5.796 |
| 300 | 4×4×1 | -7262.480775 | 2.638 |
| 320 | **4×4×1** | **-7262.520343** | **1.015** |
| 340 | 4×4×1 | -7262.53557 | 0.361 |
| 360 | 4×4×1 | -7262.540991 |  |
| 320 | 3×3×1 | -7262.37402 | 9.755 |
| 320 | **4×4×1** | **-7262.520343** | **1.041** |
| 320 | 5×5×1 | -7262.504725 | 0.815 |
| 320 | 6×6×1 | -7262.492506 |  |

**Table S3.** Convergence tests for bulk Bi_2_Te_3_.

| Cutoff energy (eV) | k-point mesh | Single-point energy (eV) | Difference (meV/atom) |
| --- | --- | --- | --- |
| 400 | 4×4×1 | -9333.433296 | 2.147 |
| 420 | 4×4×1 | -9333.465495 | 1.461 |
| 440 | **4×4×1** | **-9333.487402** | **1.041** |
| 460 | 4×4×1 | -9333.503024 | 0.739 |
| 480 | 4×4×1 | -9333.514114 |  |
| 440 | 3×3×1 | -9333.312569 | 11.656 |
| 440 | **4×4×1** | **-9333.487402** | **0.051** |
| 440 | 5×5×1 | -9333.486642 | 0.561 |
| 440 | 6×6×1 | -9333.49506 |  |

These bulk structures were then geometrically optimized to minimize internal stresses and obtain stable structures. **Figure S1a-d** display the schematic Sb_2_Te_3_ and Bi_2_Te_3_ bulk crystal structures in top-view and side-view, respectively. Sb_2_Te_3_ and Bi_2_Te_3_ have similar rhombohedral tetradymite crystal structures, both falling within the space group R-3 m H ^[1]^. They are layered materials, comprising of multiple planar Te-Sb (Bi)-Te-Sb (Bi)-Te quintuple layers connected by vdW interactions along the *c*-axis ^[2]^. The lattice constants of optimized Sb_2_Te_3_ and Bi_2_Te_3_ can be observed in **Table S4**, which are consistent with the standard database values (ICSD-193330 ^[3]^ for Bi_2_Te_3_ and ICSD-131224/CCDC Nr. 1898614 ^[4]^ for Sb_2_Te_3_). This agreement between optimized results and standard database values indicates the reliability of the DFT calculation method utilized in this work.


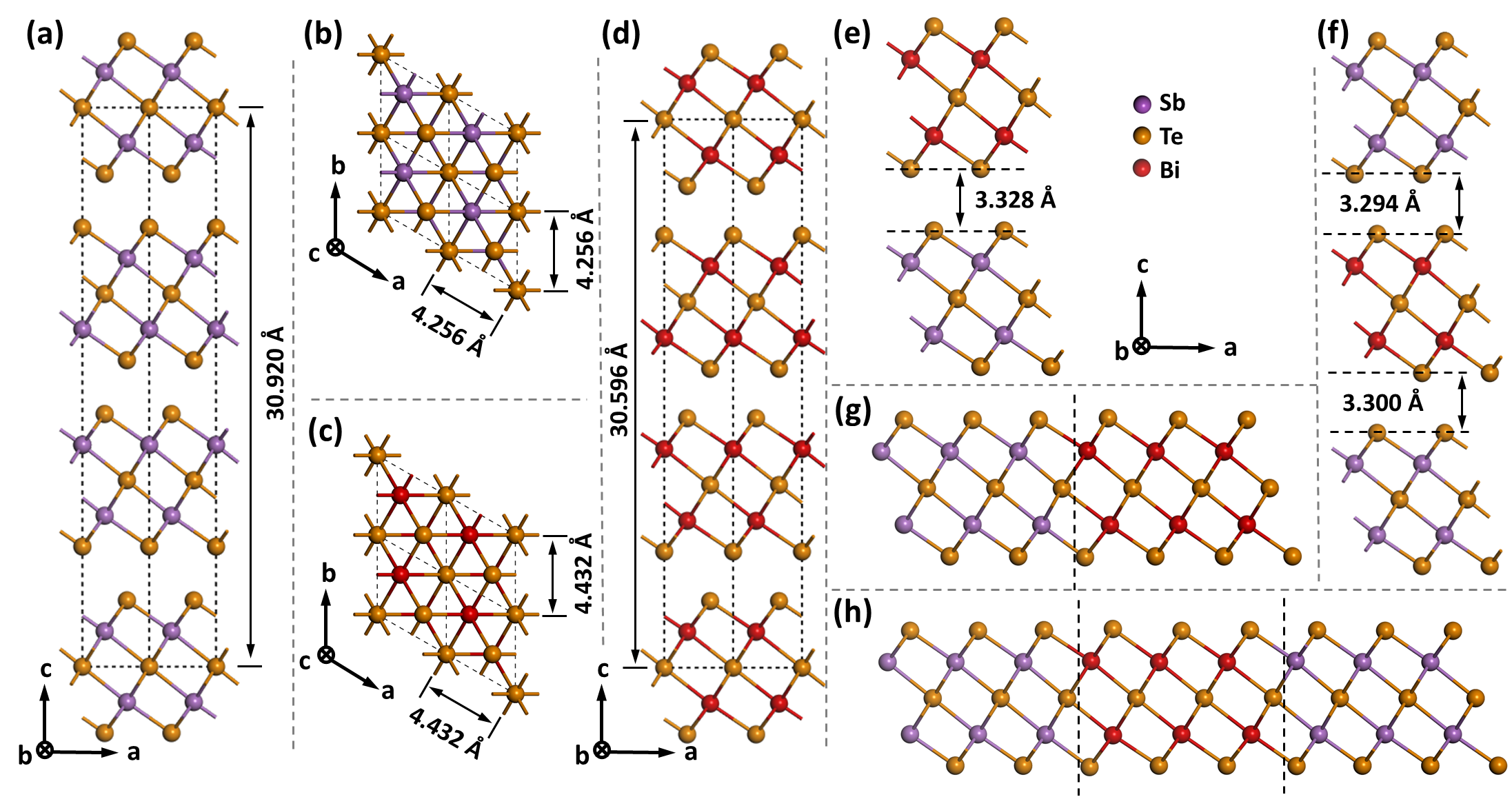


**Figure S1.** Schematic crystal structures of **(a)** Sb_2_Te_3_ bulk from side-view, **(b)** Sb_2_Te_3_ bulk from top-view, **(c)** Bi_2_Te_3_ bulk from top-view, **(d)** Bi_2_Te_3_ bulk from side-view, **(e)** Bi_2_Te_3_-Sb_2_Te_3_ vertical heterostructure, **(f)** Bi_2_Te_3_-Sb_2_Te_3_ vertical superlattice, **(g)** Bi_2_Te_3_-Sb_2_Te_3_ lateral heterostructure, and **(h)** Bi_2_Te_3_-Sb_2_Te_3_ lateral superlattice.

**Table S4.** Lattice constants of optimized Sb_2_Te_3_ bulk, Bi_2_Te_3_ bulk, quintuple-layer (QL) Sb_2_Te_3_, and QL Bi_2_Te_3_.

| Structures | *a* (Å) | *b* (Å) | *c* (Å) | *α* | *β* | *γ* |
| --- | --- | --- | --- | --- | --- | --- |
| Sb_2_Te_3_ bulk | 4.255958 | 4.255958 | 30.919865 | 90° | 90° | 120° |
| Bi_2_Te_3_ bulk | 4.431736 | 4.431736 | 30.596437 | 90° | 90° | 120° |
| QL Sb_2_Te_3_ | 4.255958 | 4.255958 | 22.39027 | 90° | 90° | 120° |
| QL Bi_2_Te_3_ | 4.431736 | 4.431736 | 22.45848 | 90° | 90° | 120° |

1. ***Lattice mismatch calculation***

The key factor for the construction of heterostructures/superlattices is the crystal lattice mismatch. The lattice mismatch can be expressed as Formula (S1), where *a_1_* and *a_2_* refer to the lattice constants of Sb_2_Te_3_ and Bi_2_Te_3_, respectively, and *a_m_* is the larger value of *a_1_* and *a_2_* ^[5]^. The lattice mismatch for Sb_2_Te_3_ and Bi_2_Te_3_ is 3.97%, 3.97%, and 1.05% along the *a*-axis, *b*-axis, and *c*-axis, respectively, which is less than 5%. This small mismatch not only enables the feasible formation of these structures but also introduces minor strains that were accounted for in our calculations ^[6]^. However, these strains have negligible effects on the electronic properties of Bi_2_Te_3_-Sb_2_Te_3_ heterostructures/superlattices due to their small magnitudes.

$$\begin{aligned} m=\left| \frac{a_{1}-a_{2}}{a_{m}} \right|\#\left( S1 \right) \end{aligned}$$

1. ***QL structure construction***

In our DFT calculations, QL Sb_2_Te_3_ and Bi_2_Te_3_ were selected as building blocks for Bi_2_Te_3_-Sb_2_Te_3_ vertical and lateral heterostructure/superlattice configurations. Similar to bulk structures, QL Sb_2_Te_3_ and Bi_2_Te_3_ were also convergence tested and geometrically optimized. The convergence tests were listed in **Table S5-S6**. 300 eV and 6 × 6 × 1 were selected as the optimal cutoff energy and k-point mesh for QL Sb_2_Te_3_, and 400 eV and 6 × 6 × 1 for QL Bi_2_Te_3_. The optimized QL Sb_2_Te_3_ and Bi_2_Te_3_ were shown in **Figure S2**, and the lattice constants of them were listed in **Table S4** as well. It should be noted that a vacuum slab of 15 Å was set to prevent interactions between adjacent QL layers in different unit cells along the *c*-axis. This thickness of the vacuum slab was determined by analyzing the work function of the Bi_2_Te_3_-Sb_2_Te_3_ vertical heterostructures/superlattices.

**Table S5.** Convergence tests for QL Sb_2_Te_3_.

| Cutoff energy (eV) | k-point mesh | Single-point energy (eV) | Difference (meV/atom) |
| --- | --- | --- | --- |
| 260 | 4×4×1 | -2420.642353 | 4.673 |
| 280 | 4×4×1 | -2420.712444 | 1.981 |
| 300 | **4×4×1** | **-2420.742152** | **0.840** |
| 320 | 4×4×1 | -2420.754758 | 0.331 |
| 340 | 4×4×1 | -2420.759717 |  |
| 300 | 4×4×1 | -2420.742152 | 4.367 |
| 300 | 5×5×1 | -2420.807652 | 3.216 |
| 300 | **6×6×1** | **-2420.759409** | **0.470** |
| 300 | 7×7×1 | -2420.766459 | 0.130 |
| 300 | 8×8×1 | -2420.768416 |  |
| 300 | **6×6×1** | **-2420.759409** | **0.004** |
| 300 | 6×6×2 | -2420.759464 | 0.006 |
| 300 | 6×6×3 | -2420.75938 |  |

**Table S6.** Convergence tests for QL Bi_2_Te_3_.

| Cutoff energy (eV) | k-point mesh | Single-point energy (eV) | Difference (meV/atom) |
| --- | --- | --- | --- |
| 360 | 4×4×1 | -3111.121814 | 1.618 |
| 380 | 4×4×1 | -3111.14608 | 1.067 |
| 400 | **4×4×1** | **-3111.16208** | **0.700** |
| 420 | 4×4×1 | -3111.172584 | 0.487 |
| 440 | 4×4×1 | -3111.179892 |  |
| 400 | 4×4×1 | -3111.16208 | 1.329 |
| 400 | 5×5×1 | -3111.182019 | 0.310 |
| 400 | **6×6×1** | **-3111.177365** | **0.680** |
| 400 | 7×7×1 | -3111.16716 | 0.759 |
| 400 | 8×8×1 | -3111.178553 |  |
| 400 | **6×6×1** | **-3111.177365** | **0.003** |
| 400 | 6×6×2 | -3111.177315 | 0.001 |
| 400 | 6×6×3 | -3111.177329 |  |


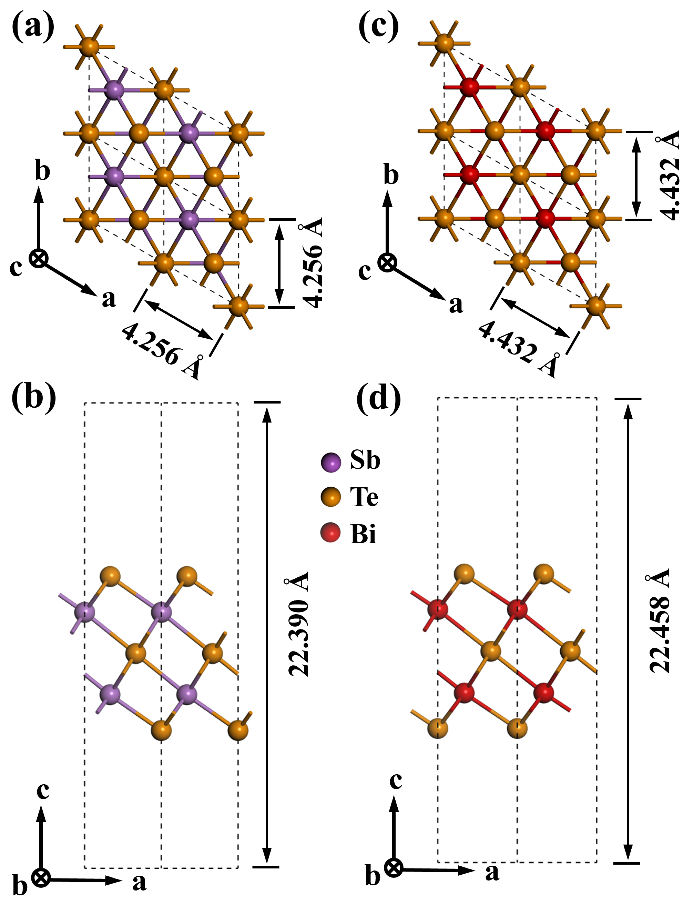


**Figure S2.** Schematic crystal structures of **(a)** QL Sb_2_Te_3_ from top-view, **(b)** QL Sb_2_Te_3_ from side-view, **(c)** QL Bi_2_Te_3_ from top-view, and **(d)** QL Bi_2_Te_3_ from side-view.

1. ***Vertical heterostructure/superlattice construction***

The "Build layers" tool in Materials Studio was used to create initial configurations of Bi_2_Te_3_-Sb_2_Te_3_ vertical heterostructures/superlattices. However, these initial setups required further refinement before DFT calculations. It should be noted that average lattice constants of the constituent QL Sb_2_Te_3_ and Bi_2_Te_3_ were used to construct the heterostructures/ superlattices.

1. ***Structural refinement for vertical heterostructures/superlattices***

A three-step process was implemented to determine the most stable configuration (minimal energy): (1) The interlayer distance (***d***) between QLs along the *c*-axis was systematically adjusted, and single-point energy calculations were performed for each configuration to identify the lowest energy state, as shown in **Figure S3a** and **Figure S4a**; (2) Either QL Sb_2_Te_3_ or QL Bi_2_Te_3_ was displaced along the *a*-axis (***d_a_***), with single-point energy calculations for each position (**Figure S3b** and **Figure S4b**); (3) Similar to the *a*-axis, displacements and energy calculations were performed along the *b*-axis (***d_b_***), as depicted in **Figure S3c** and **Figure S4c**.

A similar approach was applied to the superlattice configurations. The interlayer distances between successive QLs were varied (***d_1_*** and ***d_2_***), and single-point energy calculations were performed to determine the most stable stacking arrangement, as depicted in **Figure S3d** and **Figure S4d**. The relative displacements along the *a*- and *b*-axes were not redefined, as they were previously optimized in the heterostructure calculations.


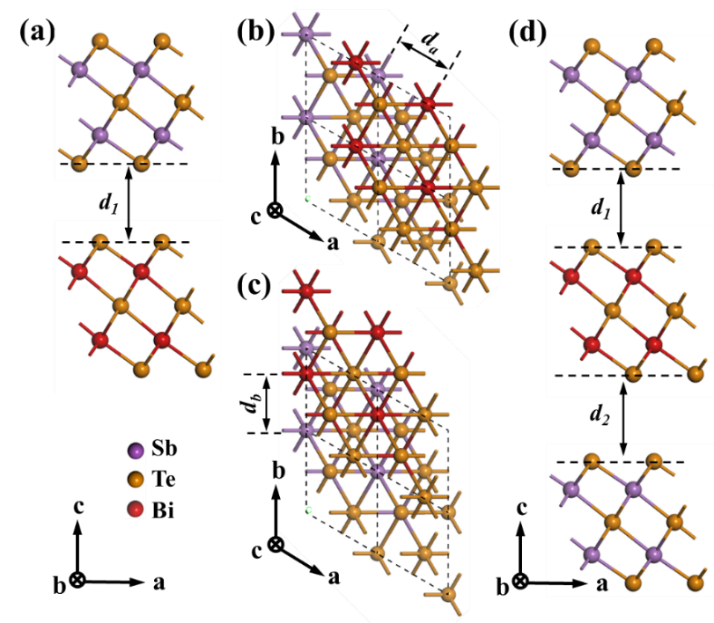


**Figure S3.** Schematic diagrams of structural refinement for Bi_2_Te_3_-Sb_2_Te_3_ vertical (a)-(c) heterostructures and (d) superlattices.


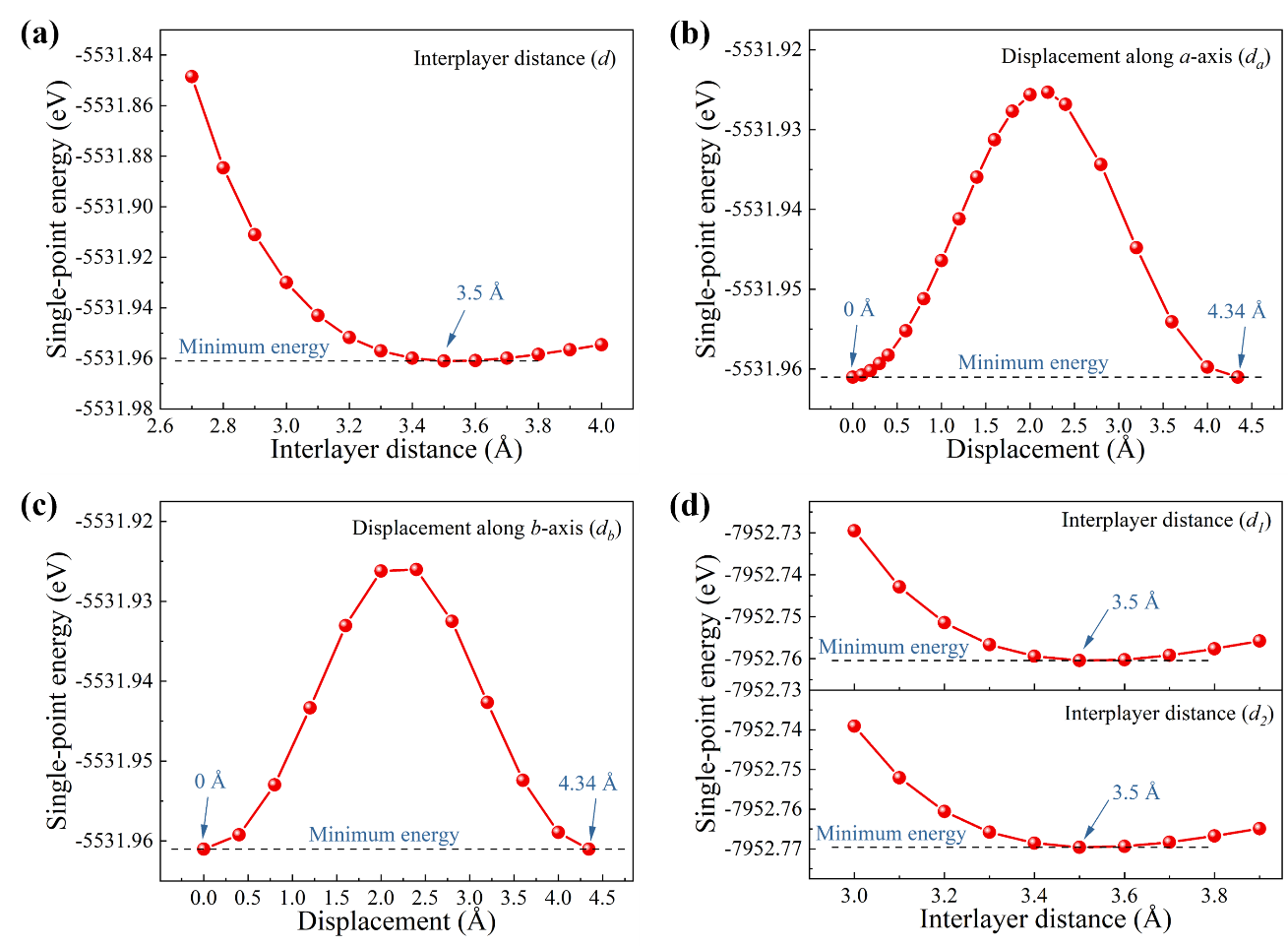


**Figure S4.** Single-point energy *versus* (a) interlayer distance (*d*), (b) lateral displacement along the a-axis (*d_a_*), and (c) lateral displacement along the b-axis (*d_b_*) curves of Bi_2_Te_3_-Sb_2_Te_3_ vertical heterostructures; (d) single-point energy *versus* interlayer distances (*d_1_* and *d_2_*) curves of Bi_2_Te_3_-Sb_2_Te_3_ vertical superlattice.

1. ***Lateral heterostructure/superlattice construction***

For lateral configurations, we assumed intra-planar covalent bonding between QL Sb_2_Te_3_ and QL Bi_2_Te_3_ on the (1 0 0) plane. Interestingly, subsequent experimental observations *via* TEM imaging of the lateral heterostructures interface revealed the interface along the (3 0 0) plane, indicating a strong correlation between our theoretical assumptions and experimental results. This alignment highlights the reliability of our modelling and DFT calculations. The intra-planar heterostructures/superlattices were created by first building a supercell of one material (e.g., 6 × 1 and 9 × 1 unit-cell of QL Sb_2_Te_3_) and then substituting specific atoms to create the desired heterostructure or superlattice. The fundamental building blocks in these structures consist of 3 × 1 unit-cells of QL Sb_2_Te_3_ and 3 × 1 unit-cells of QL Bi_2_Te_3_, with covalent bonding across the interfaces, as shown in **Figure S5**. The choice of 3 × 1 unit-cells for each material component in our lateral heterostructures and superlattices ensures a surface thickness exceeding 10 Å. This thickness is crucial for maintaining bulk-like behavior in central layers, minimizing artificial interactions in periodic DFT calculations, allowing proper electronic and structural relaxation, and ensuring accurate interfacial energetics.


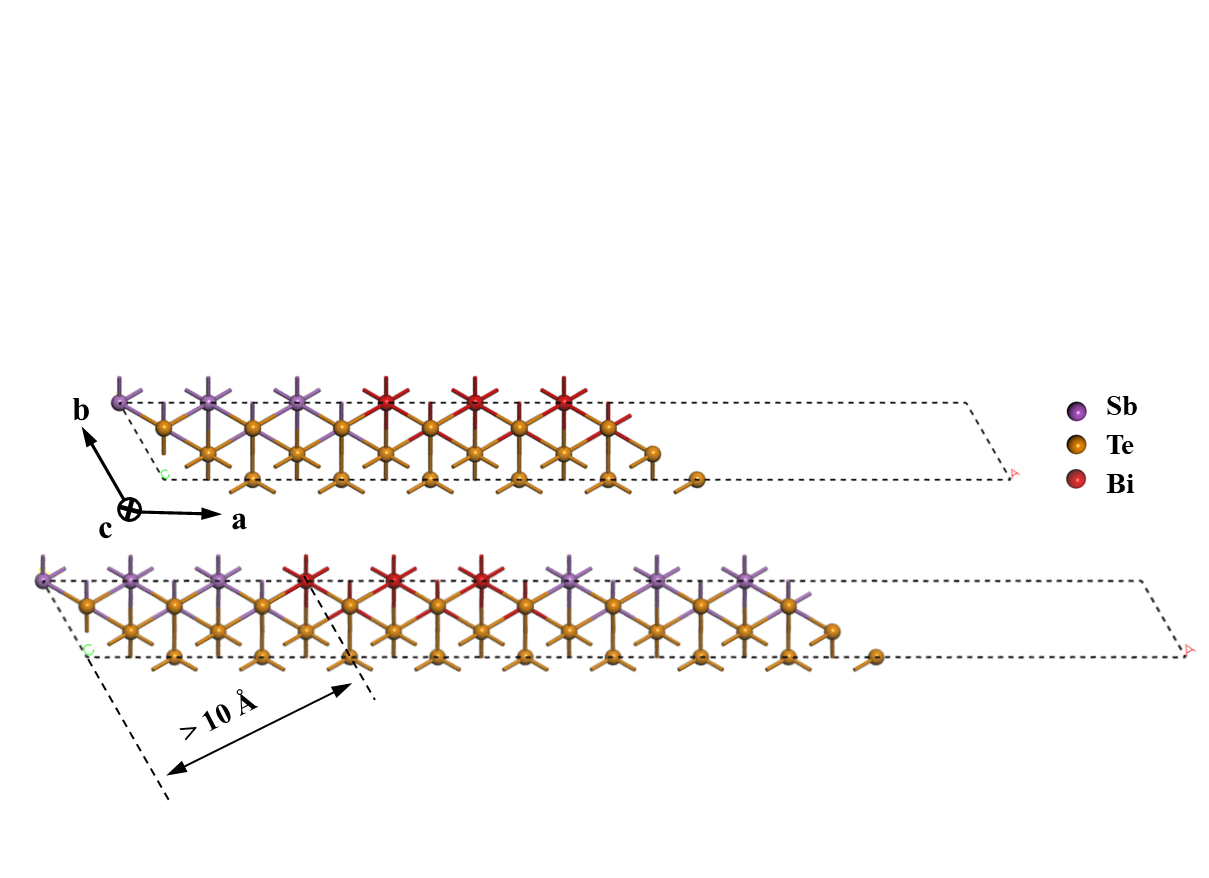


**Figure S5.** Schematic diagrams of Bi_2_Te_3_-Sb_2_Te_3_ vertical heterostructures (upper) and superlattices (bottom).

1. ***Final convergence tests and optimization***

After determining the initial configurations, convergence tests were performed to establish the appropriate cutoff energy and k-point mesh, as detailed in **Table S7-S10**. Based on these tests, we determined optimal cutoff energies of 400 eV for vertical structures and 440 eV for lateral structures. For k-point mesh, we used Monkhorst-Pack grids of 6 × 6 × 1 for vertical structures and 1 × 6 × 1 for lateral structures. These structures were then fully geometrically optimized before conducting the interface energy calculations to ensure all results were obtained from a stable configuration. The optimized Bi_2_Te_3_-Sb_2_Te_3_ vertical and lateral heterostructures/superlattices were depicted in **Figure S1**.

**Table S7.** Convergence tests for 3 × 1 unit-cells of QL Sb_2_Te_3_.

| Cutoff energy (eV) | k-point mesh | Single-point energy (eV) | Difference (meV/atom) |
| --- | --- | --- | --- |
| 300 | 1×5×1 | -7259.466221 | 3.493 |
| 300 | 1×6×1 | -7259.413831 | 0.498 |
| 300 | 1×7×1 | -7259.406359 | 1.580 |
| 300 | 1×8×1 | -7259.43005 |  |
| 300 | **1×6×1** | **-7259.413831** | **0.001** |
| 300 | 2×6×1 | -7259.413841 | 0.006 |
| 300 | 6×3×1 | -7259.413746 |  |
| 300 | **1×6×1** | **-7259.413831** | **0.005** |
| 300 | 2×6×2 | -7259.413909 | 0.013 |
| 300 | 1×6×3 | -7259.414102 |  |
| 280 | 1×6×1 | -7259.322684 | 6.076 |
| 300 | 1×6×1 | -7259.413831 | 2.530 |
| 320 | **1×6×1** | **-7259.451774** | **0.979** |
| 340 | 1×6×1 | -7259.466449 | 0.345 |
| 360 | 1×6×1 | -7259.471623 |  |

**Table S8.** Convergence tests for 3 × 1 unit-cells of QL Bi_2_Te_3_.

| Cutoff energy (eV) | k-point mesh | Single-point energy (eV) | Difference (meV/atom) |
| --- | --- | --- | --- |
| 400 | 1×5×1 | -9329.991354 | 1.404 |
| 400 | 1×6×1 | **-9329.970287** | **0.706** |
| 400 | 1×7×1 | -9329.959702 | 1.433 |
| 400 | 1×8×1 | -9329.981198 |  |
| 400 | **1×6×1** | **-9329.970287** | **0.001** |
| 400 | 2×6×1 | -9329.970276 | 0.001 |
| 400 | 6×3×1 | -9329.970254 |  |
| 400 | **1×6×1** | **-9329.970287** | **0.004** |
| 400 | 2×6×2 | -9329.970346 | 0.006 |
| 400 | 1×6×3 | -9329.97043 |  |
| 400 | 1×6×1 | -9329.970287 | 2.177 |
| 420 | 1×6×1 | -9330.002945 | 1.425 |
| 440 | **1×6×1** | **-9330.024323** | **0.992** |
| 460 | 1×6×1 | -9330.039201 | 0.700 |
| 480 | 1×6×1 | -9330.049698 |  |

**Table S9.** Convergence tests for Bi_2_Te_3_-Sb_2_Te_3_ vertical heterostructures/superlattices.

| Cutoff energy (eV) | k-point mesh | Single-point energy (eV) | Difference (meV/atom) |
| --- | --- | --- | --- |
| Bi_2_Te_3_-Sb_2_Te_3_ vertical heterostructures | | | |
| 380 | 6×6×1 | -5531.944979 | 1.064 |
| 400 | **6×6×1** | **-5531.960944** | **0.730** |
| 420 | 6×6×1 | -5531.971895 | 0.502 |
| 440 | 6×6×1 | -5531.979425 | 0.343 |
| 460 | 6×6×1 | -5531.984573 |  |
| Bi_2_Te_3_-Sb_2_Te_3_ vertical superlattices | | | |
| 380 | 6×6×1 | -7952.753233 | 1.087 |
| 400 | **6×6×1** | **-7952.769544** | **0.737** |
| 420 | 6×6×1 | -7952.780603 | 0.509 |
| 440 | 6×6×1 | -7952.788234 | 0.349 |
| 460 | 6×6×1 | -7952.793474 |  |

**Table S10.** Convergence tests for Bi_2_Te_3_-Sb_2_Te_3_ lateral heterostructures/superlattices.

| Cutoff energy (eV) | k-point mesh | Single-point energy (eV) | Difference (meV/atom) |
| --- | --- | --- | --- |
| Bi_2_Te_3_-Sb_2_Te_3_ lateral heterostructures | | | |
| 400 | 1×6×1 | -16589.86283 | 2.154 |
| 420 | 1×6×1 | -16589.89514 | 1.557 |
| 440 | **1×6×1** | **-16589.91851** | **1.135** |
| 460 | 1×6×1 | -16589.93553 | 0.852 |
| 480 | 1×6×1 | -16589.94831 |  |
| Bi_2_Te_3_-Sb_2_Te_3_ lateral superlattices | | | |
| 420 | 1×6×1 | -23852.3919 | 2.053 |
| 440 | **1×6×1** | **-23852.42269** | **1.347** |
| 460 | 1×6×1 | -23852.4429 | 0.925 |
| 480 | 1×6×1 | -23852.45677 |  |

### (2) Interface Energy Calculations

In order to evaluate the thermodynamic stability of Bi_2_Te_3_-Sb_2_Te_3_ vertical and lateral heterostructure/superlattice, the interface forming energies were calculated by Formula (S2) and Formula (S3), where $S$ represents the total interfacial area of the heterostructure/superlattice, $E({Sb}_{2}{Te}_{3})$, $E({Bi}_{2}{Te}_{3})$, $E(HS)$, and $E(SL)$ are the total energies of QL Sb_2_Te_3_, QL Bi_2_Te_3_, their heterostructure, and their superlattice, respectively ^[7]^.

$$\begin{aligned} \Delta E(HS)=\frac{E({Sb}_{2}{Te}_{3})+E({Bi}_{2}{Te}_{3})-E(HS)}{S}\#\left( S2 \right) \end{aligned}$$

$$\begin{aligned} \Delta E(SL)=\frac{2E({Sb}_{2}{Te}_{3})+E({Bi}_{2}{Te}_{3})-E(SL)}{S}\#\left( S3 \right) \end{aligned}$$

The calculated interface forming energies of Bi_2_Te_3_-Sb_2_Te_3_ heterostructure/superlattice were listed in **Table 2**. These values are positive and smaller than previously reported data of corresponding structures, such as BiOI-BiOIO_3_ vertical heterostructure (8 meV Å^-2^) ^[6]^, g-C_3_N_4_-BiOI vertical heterostructure (10.96 meV Å^-2^) ^[7]^, Hf_2_CO_2_-GaN vertical heterostructure (52.44 meV Å^-2^) ^[8]^, GeS-SnS lateral heterostructure (210 meV Å^-2^) ^[9]^, In_2_STe-InSe lateral heterostructure (110 and 180 meV Å^-2^) ^[10]^, GeS-GeSe lateral heterostructure (290 meV Å^-2^) ^[9]^, etc. Since this DFT calculation has promised as a viable method to predict other 2D heterostructures previously ^[11]^, these interface face calculations and comparison indicate that it is highly feasible to grow Bi_2_Te_3_-Sb_2_Te_3_ heterostructure/superlattice with high stability.

In our calculations, the interface energies of vertical structures are much lower than those of lateral structures, which can be attributed to key physical differences in bonding types at the interface. In vertical heterostructures/superlattices, the QLs are primarily held together by weak vdW interactions. In contrast, lateral heterostructures involve stronger covalent bonding at the interface. The covalent bonding increases the interface energy significantly compared to vertical structures.

## Characterization


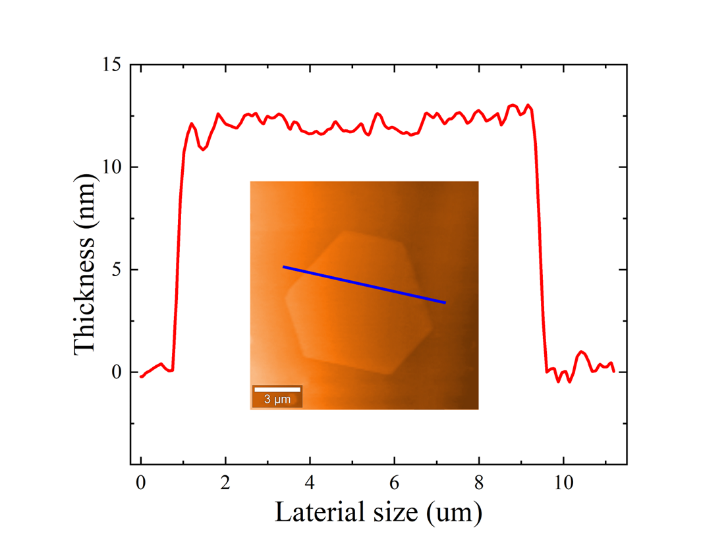


**Figure S6** Height profile (along the blue line on the inset) with the AFM image of 2D vdW Bi_2_Te_3_-Sb_2_Te_3_ superlattices.


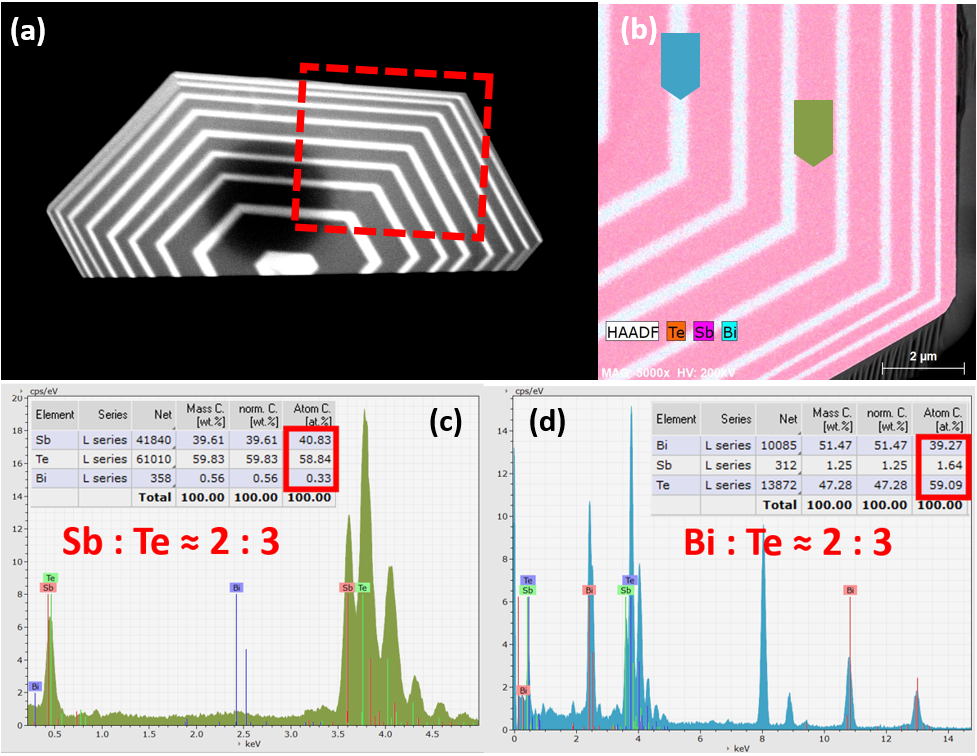


**Figure S7. (a)** Top-view SEM image with in-column detector (ICD) of a representative 17-layer 2D vdW superlattices and **(b)** HAADF-STEM image and EDX mapping with chemical ratio for **(c)** Sb_2_Te_3_ layer (green indicator and spectrum) and **(d)** Bi_2_Te_3_ layer (blue indicator and spectrum).


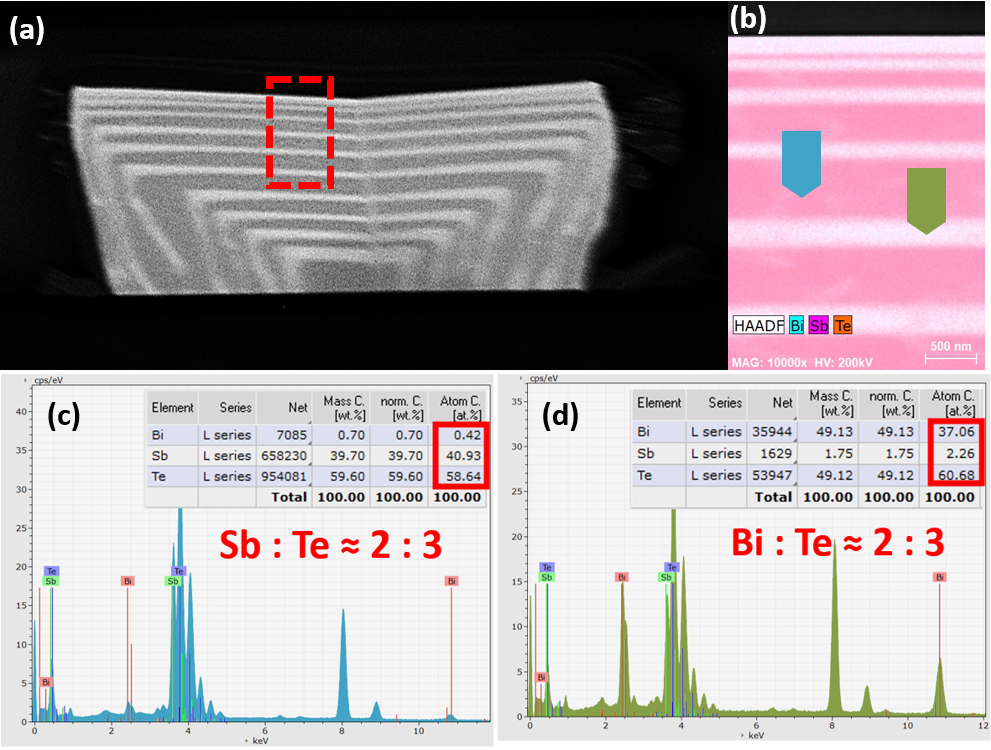


**Figure S8. (a)** Side-view SEM image with ICD of a representative 19-layer bulk vdW superlattices and **(b)** HAADF-STEM image and EDX mapping with chemical ratio for **(c)** Sb_2_Te_3_ layer (blue indicator and spectrum) and **(d)** Bi_2_Te_3_ layer (green indicator and spectrum).


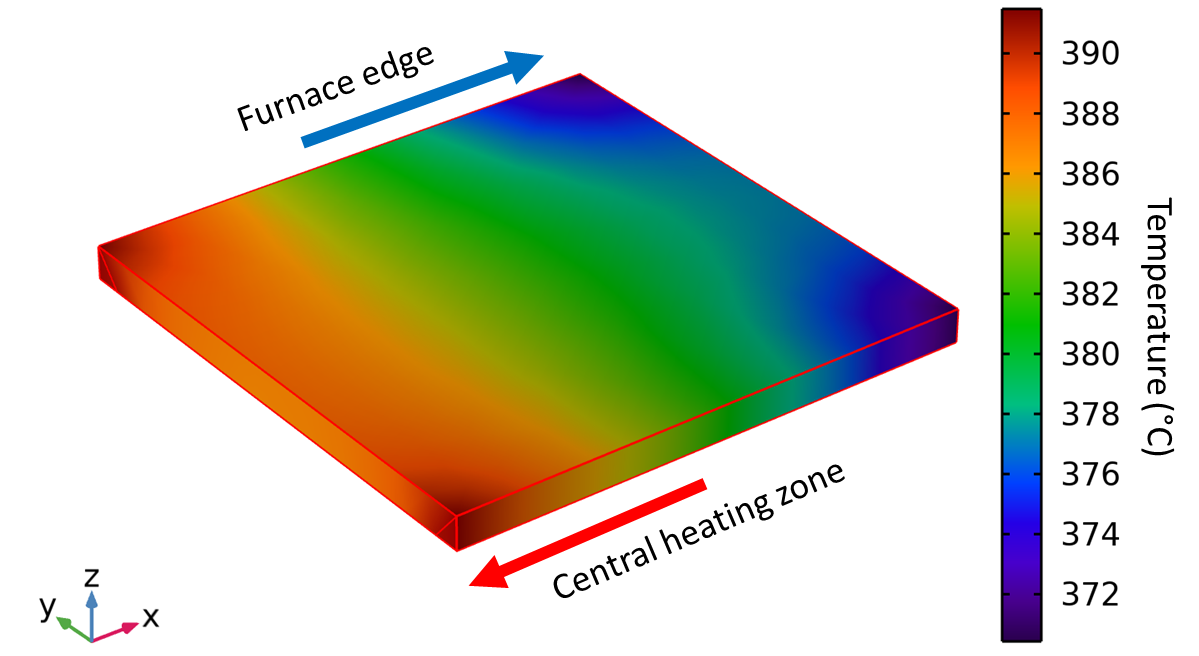


**Figure S9.** Growth temperature distribution on the SiO_2_ substrate surface for 2D vdW superlattices modelled with ANSYS thermodynamic analysis. The precursor temperature was set to 500°C. The leading edge of the substrate was positioned 13.5 cm downstream from the central heating zone. The size of the single SiO_2_ substrate is 1 cm × 1 cm × 15 µm.


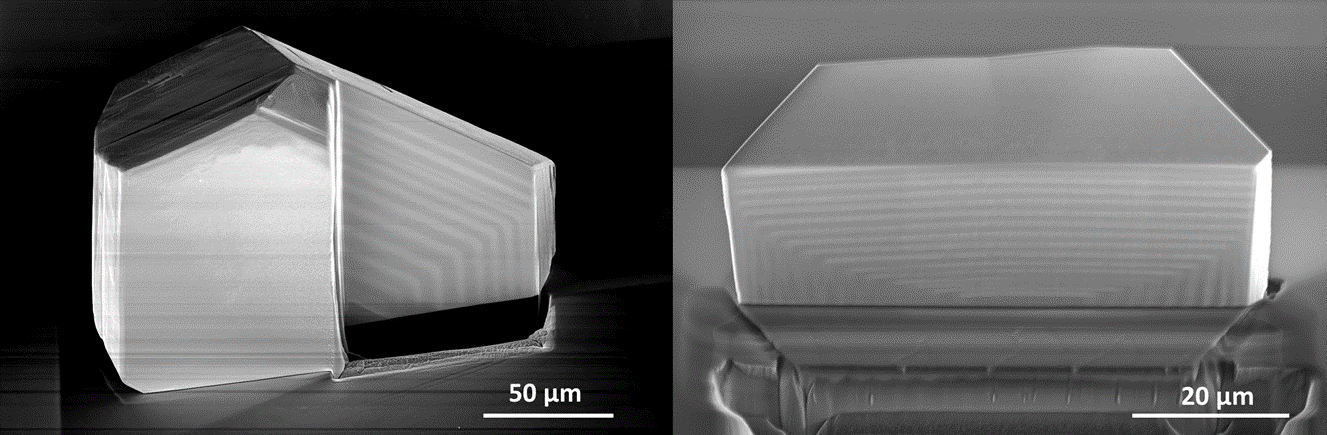


**Figure S10.** SEM images for the transverse (left) and longitudinal (right) cross-sections of a representative 32-layer Bi_2_Te_3_-Sb_2_Te_3_ superlattice, respectively.


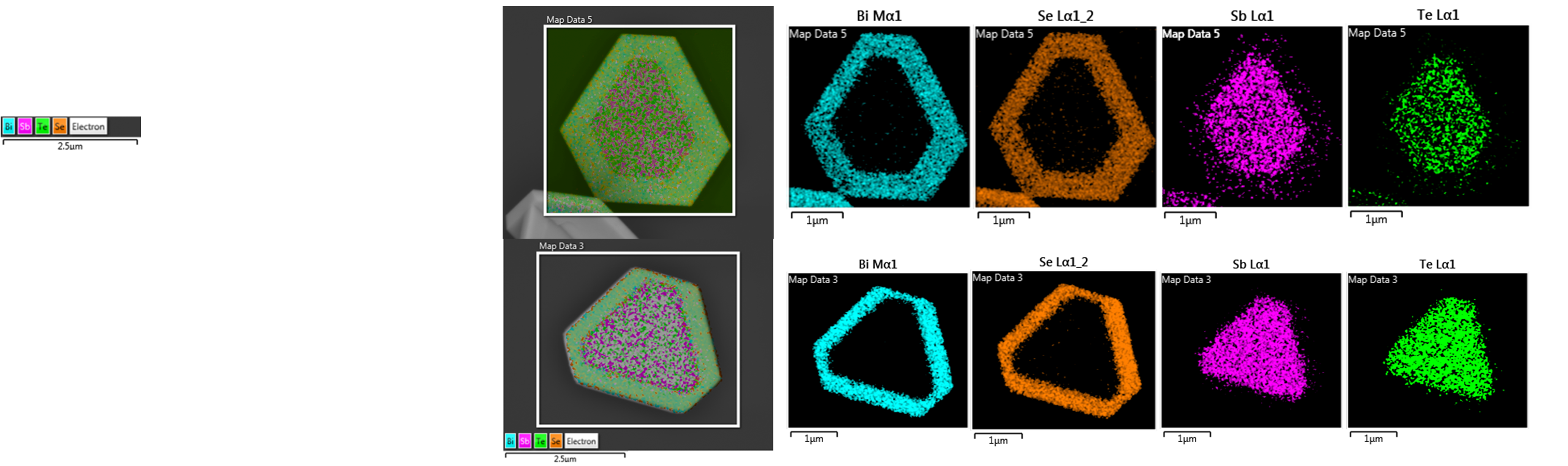


**Figure S11.** Full-scale EDX mapping of 2-layer lateral 2D vdW Bi_2_Se_3_/Sb_2_Te_3_ heterostructure and the corresponding elemental distributions for Bi, Se, Sb and Te.

## Operational Procedures, Scalability and Uniformity of FIB Technique

The impact of the FIB cutting process on the structural and electronic properties of the superlattices is minimal due to well-established standard operating procedure developed for TEM specimen preparation. FIB is widely used in TEM specimen preparation, where the requirements for sample thickness and preservation of crystal quality are extremely stringent. Typically, TEM specimens must be less than 100 nm thick without damaging the crystal quality of the cross-section. As a result, a comprehensive set of procedures has been developed to ensure that the ion beam does not damage the sample. Firstly, a thin carbon layer (~50 nm) of electron beam deposition is applied to protect the top surface of the sample. This electron beam deposition is preferred over ion beam deposition as it has less impact on the sample surface. After the initial cutting, the sample cross-section is polished using low voltage and low current to ensure the preservation of crystal quality at the surface. In our study, we followed these established operating procedures for all FIB-based operations, except when deliberately deconstructing the superlattice layer by layer (**Figure 8d**). These protective measures effectively shield the material from ion beam damage, preserving the intrinsic characteristics of the superlattice structure.

About the scalability and uniformity of the FIB cutting method, especially for larger samples, we acknowledge that FIB cutting limits its throughput for large-scale production. The time required for cutting increases linearly with the sample size, making it challenging for industrial-scale applications. However, this is a preliminary work, and we are investigating the feasibility of industrial-scale methods for fabricating these vertical superlattices. Specifically, we are considering standard industry approach such as reactive ion etching and nanoimprint lithography for larger scale fabrication which can well address scalability and uniformity challenges confronted by FIB cutting. This present as an important topic for future study.

## Device Fabrication and Characterization


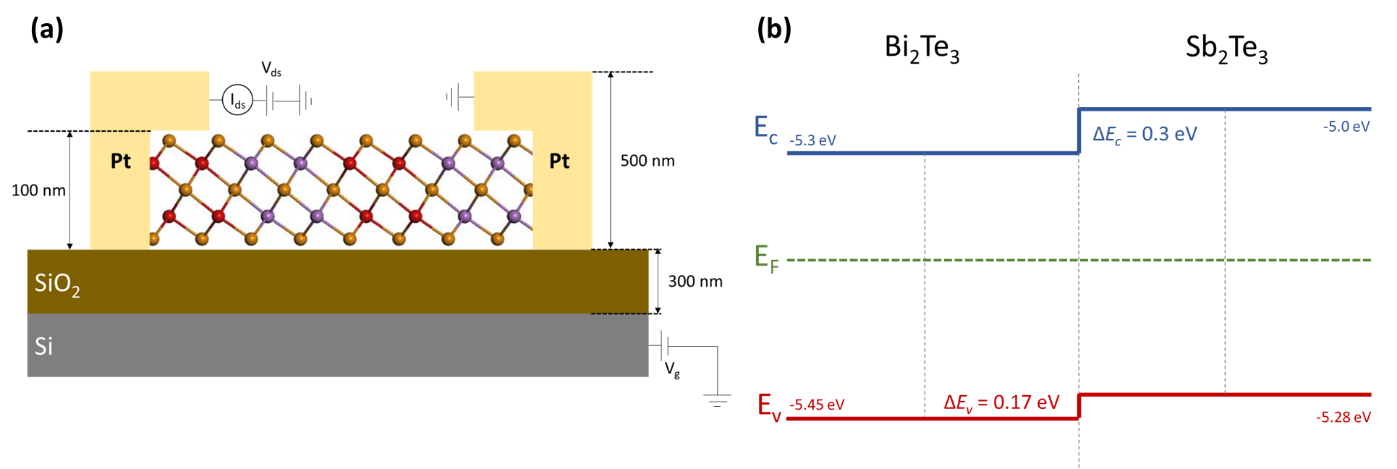


**Figure S12. (a)** Cross-sectional schematic of the Bi_2_Te_3_-Sb_2_Te_3_ FET structure with electrical connections used to characterize the device. **(b)** Schematic band structure of the achieved Bi_2_Te_3_-Sb_2_Te_3_ superlattices, indicating type-II band alignment.

**Table S11.** Main growth parameters for the growth of 2D vdW superlattices.

| Tube inner pressure | 3 Torr |
| --- | --- |
| Carrier gas flow rate | 80 sccm (standard cubic centimeters per minute) |
| Heating rate | 25°C/minute |
| Default reaction period | 30 minutes for each step |
| Interval time | 5 minutes |
| Precursor temperature | melting point for each precursor |

## Reference

[1] P. Guha, J. Y. Park, J. Jo, Y. Chang, H. Bae, R. K. Saroj, H. Lee, M. Kim, G.-C. Yi, *2d Mater* **2022**, 9, 025006.

[2] a) S. R. Bhopale, M. A. More, *physica status solidi (a)* **2022**, 219, 2200126; b) H. Bryja, J. W. Gerlach, A. Prager, M. Ehrhardt, B. Rauschenbach, A. Lotnyk, *2d Mater* **2021**, 8, 045027.

[3] A. N. Mansour, W. Wong-Ng, Q. Huang, W. Tang, A. Thompson, J. Sharp, *J Appl Phys* **2014**, 116, 083513.

[4] M. Kuepers, R. P. Stoffel, B. Bong, M. G. Herrmann, Z. K. Li, A. Meledin, J. Mayer, K. Friese, R. Dronskowski, *Z Naturforsch B* **2020**, 75, 41.

[5] G. Guo, C. Xu, S. Tan, Z. Xie, *Physica E: Low-dimensional Systems and Nanostructures* **2022**, 143, 115359.

[6] W. W. Dai, Z. Y. Zhao, *Physical Chemistry Chemical Physics* **2017**, 19, 9900.

[7] W. W. Dai, Z. Y. Zhao, *Applied Surface Science* **2017**, 406, 8.

[8] K. Ren, R. X. Zheng, P. Xu, D. Cheng, W. Y. Huo, J. Yu, Z. R. Zhang, Q. Y. Sun, *Nanomaterials-Basel* **2021**, 11.

[9] K. Cheng, Y. Guo, N. N. Han, Y. Su, J. F. Zhang, J. J. Zhao, *Journal of Materials Chemistry C* **2017**, 5, 3788.

[10] G. Guo, C. S. Xu, S. Y. Tan, Z. X. Xie, *Physica E* **2022**, 143.

[11] a) Y. J. Gong, J. H. Lin, X. L. Wang, G. Shi, S. D. Lei, Z. Lin, X. L. Zou, G. L. Ye, R. Vajtai, B. I. Yakobson, H. Terrones, M. Terrones, B. K. Tay, J. Lou, S. T. Pantelides, Z. Liu, W. Zhou, P. M. Ajayan, *Nature Materials* **2014**, 13, 1135; b) X. D. Duan, C. Wang, J. C. Shaw, R. Cheng, Y. Chen, H. L. Li, X. P. Wu, Y. Tang, Q. L. Zhang, A. L. Pan, J. H. Jiang, R. Q. Yu, Y. Huang, X. F. Duan, *Nat Nanotechnol* **2014**, 9, 1024; c) S. Najmaei, Z. Liu, W. Zhou, X. L. Zou, G. Shi, S. D. Lei, B. I. Yakobson, J. C. Idrobo, P. M. Ajayan, J. Lou, *Nature Materials* **2013**, 12, 754.

1. *Corresponding authors. 1,*) E-mail: [wen.lei@uwa.edu.au](mailto:wen.lei@uwa.edu.au), 2,*) E-mail: [liujunliang@jiangnan.edu.cn](mailto:liujunliang@jiangnan.edu.cn),^a^ These authors contributed to the work equally. [↑](#footnote-ref-2)
2. [↑](#footnote-ref-3)
